# Sequence variants affecting voice pitch in humans

**DOI:** 10.1126/sciadv.abq2969

**Published:** 2023-06-09

**Authors:** Rosa S. Gisladottir, Agnar Helgason, Bjarni V. Halldorsson, Hannes Helgason, Michal Borsky, Yu-Ren Chien, Jon Gudnason, Sigurjon A. Gudjonsson, Scott Moisik, Dan Dediu, Gudmar Thorleifsson, Vinicius Tragante, Mariana Bustamante, Gudrun A. Jonsdottir, Lilja Stefansdottir, Gudrun Rutsdottir, Sigurdur H. Magnusson, Marteinn Hardarson, Egil Ferkingstad, Gisli H. Halldorsson, Solvi Rognvaldsson, Astros Skuladottir, Erna V. Ivarsdottir, Gudmundur Norddahl, Gudmundur Thorgeirsson, Ingileif Jonsdottir, Magnus O. Ulfarsson, Hilma Holm, Hreinn Stefansson, Unnur Thorsteinsdottir, Daniel F. Gudbjartsson, Patrick Sulem, Kari Stefansson

**Affiliations:** ^1^ deCODE Genetics/Amgen Inc., Sturlugata 8, 101 Reykjavik, Iceland.; ^2^ Department of Icelandic and Comparative Cultural Studies, University of Iceland, Saemundargata 2, 102 Reykjavik, Iceland.; ^3^ Department of Anthropology, University of Iceland, Saemundargata 10, 102 Reykjavik, Iceland.; ^4^ Department of Engineering, Reykjavik University, Menntavegur 1, 101 Reykjavik, Iceland.; ^5^ Division of Linguistics and Multilingual Studies, Nanyang Technological University, 50 Nanyang Avenue, Singapore 639798, Singapore.; ^6^ Department of Catalan Philology and General Linguistics, University of Barcelona, Gran Via 585, Barcelona 08007, Spain.; ^7^ University of Barcelona Institute for Complex Systems (UBICS), Martí Franquès 1, Barcelona 08028, Spain.; ^8^ Catalan Institute for Research and Advanced Studies (ICREA), Passeig Lluís Companys 23, Barcelona 08010, Spain.; ^9^ School of Engineering and Natural Sciences, University of Iceland, Dunhagi 5, 107 Reykjavik, Iceland.; ^10^ Faculty of Medicine, University of Iceland, Vatnsmyrarvegur 16, 101 Reykjavik, Iceland.

## Abstract

The genetic basis of the human vocal system is largely unknown, as are the sequence variants that give rise to individual differences in voice and speech. Here, we couple data on diversity in the sequence of the genome with voice and vowel acoustics in speech recordings from 12,901 Icelanders. We show how voice pitch and vowel acoustics vary across the life span and correlate with anthropometric, physiological, and cognitive traits. We found that voice pitch and vowel acoustics have a heritable component and discovered correlated common variants in *ABCC9* that associate with voice pitch. The *ABCC9* variants also associate with adrenal gene expression and cardiovascular traits. By showing that voice and vowel acoustics are influenced by genetics, we have taken important steps toward understanding the genetics and evolution of the human vocal system.

## Teaser

A genome-wide association study of speech acoustics in 12,901 Icelanders identified a genetic locus for voice pitch.

## INTRODUCTION

Humans are a speaking species. While nonhuman primates favor manual and bodily gestures for many aspects of social communication, during the course of evolution, our ancestors shifted to the vocal channel as the primary medium for language (*1*, *2*). Genetic studies of the human vocal system have mainly focused on voice or speech disorders and syndromes (*3*–*5*) or investigated species such as songbirds or vocal learning bats for insights into speech (*6*, *7*). Here, we turn to the vocal signals themselves, investigating the genetics of voice and vowel acoustics obtained from speech recordings of 12,901 Icelanders. Acoustic measures of speech provide insights into the genetics of the human vocal system, as they are sensitive to factors affecting the larynx and other structures that generate the sound of our speech (*8*).

The acoustics of speech sounds like vowels and consonants are described by source-filter theory (*8*, *9*). Voiced sounds are produced by the vibration of the vocal folds (the voice source). The articulatory movements of the tongue, lips, jaw, and other structures (the vocal tract filter) alter the shape of the vocal tract and its filtering effect on the voice source, resulting in a spectral profile that is characteristic for each speech sound (*8*, *9*).

An essential voice source component is voice pitch, i.e., how deep or high the voice sounds. Voice pitch is the perceptual correlate of fundamental frequency or *f*_o_ [also known as f0 or F0 (*10*)], which reflects the rate of vocal fold vibration. Henceforth, we refer to *f*_o_ as voice pitch. Humans manipulate voice pitch not only to express emotions and convey linguistic information (e.g., questions in most languages, and complex semantic and grammatical distinctions in tone languages such as Mandarin Chinese) but also to exaggerate perceptions of body size and other characteristics (*11*–*13*). Average (or habitual) voice pitch is reported to correlate with evolutionarily and socially important attributes such as hormone profiles, physical strength, reproductive success, and perceptions of attractiveness, masculinity/femininity, and dominance (*14*–*19*). For instance, studies indicate that men with deeper voices may have higher levels of testosterone and more upper-body strength (*16*, *17*), father more children (in a sample of hunter-gatherers) (*19*), are judged to be more attractive and dominant (*16*, *20*), and manage larger businesses (*21*). Voice pitch in males is about 5 standard deviations (SD) below the average female voice—a difference that far exceeds potential influences of body size (*17*). The extreme sexual dimorphism in voice pitch—greater than in any other ape (*17*)—has led some to propose that voice pitch has undergone sexual selection in males (*17*, *22*).

Another voice source measure is voice pitch variability, often measured with the SD of *f*_o_ (*f*_o_ SD). Voice pitch variability captures intonation in speech, including monotone or exaggerated pitch. Like average voice pitch, variability in voice pitch could have an impact on mate choice in humans (*15*), but its physiological and perceptual correlates are not as well understood.

The primary measures of the vocal tract filter are known as formants (labeled *F*_1_, *F*_2_, and so on). These are the vocal tract resonant frequencies that play a key role in distinguishing vowels (e.g., the vowel in “meet” from “met”). The formant structure of a vowel is determined by vocal tract shape, which is in turn influenced by speech movements (*8*). Formants have played a prominent role in speech evolution research, as they are an important ingredient in spoken language and easy to simulate in computational models (*23*). Vowel formants are the most reliable vocal cue of body size in humans (*24*) and can be modulated by speakers to amplify perceptions of size and masculinity/femininity (*13*), highlighting a social function beyond the linguistic content.

In summary, voice and vowel measures are important means of interpersonal communication, at both the linguistic and nonlinguistic levels, with clear social relevance and potential evolutionary importance. As a bonus, such measures are also useful from a clinical perspective. In particular, vowel formants and aggregated vowel measures are sensitive to neurological factors affecting speech, including speech abnormalities observed in Parkinson’s disease (*25*).

Twin studies indicate that there are genetic influences on voice pitch and other voice characteristics (*26*–*28*). Various vocal tract components are also heritable (*29*–*31*), raising the possibility that vowel formants—while clearly influenced by culture and context—might have a genetic component, because vowel formants are affected by the vocal tract. However, as large-scale genetic studies of these measures have not been conducted to date, their heritability as calculated from single-nucleotide polymorphisms (SNPs) is unknown.

Here, we perform a genome-wide association study (GWAS) of voice and vowel acoustics based on speech recordings from 12,901 Icelanders with genotypes. Iceland is an ideal research site for this purpose because it is genetically and linguistically homogeneous with minor dialectal variation (*32*). Our aim was to identify sequence variants associated with diversity in voice and speech, and thereby some of the genes and other functional elements behind the common genetic architecture of the vocal system in humans. Given the potential evolutionary importance of voice and vowel acoustics, we first explore how voice pitch and vowel formants vary across the life span and correlate with anthropometric, physiological, and cognitive traits, making use of over 20,000 additional phenotypes available for the participants of this study. By adopting a hypothesis-free approach, we are not limited by expectations based on prior work using smaller samples. We then estimate the SNP-based heritability of voice and vowel measures. Last, we perform a GWAS for insights into the biological mechanisms affecting these traits, leading to the discovery of the first genetic locus for voice pitch in humans. Together, our findings offer a window into the phenotypic and genetic architecture of the human vocal system.

## RESULTS

### Voice and vowel acoustics

We obtained speech recordings in Iceland as a part of comprehensive phenotyping of a general population sample (*33*). Participants performed a combination of speech elicitation tasks: production of isolated vowels, a sustained [a] ("ah"-sound sustained for a duration of about 4 s), single words, and read speech. We could compute acoustic measures for 12,901 individuals (total participants *N* = 14,144). For voice pitch, we estimated voice pitch with the median *f*_o_ in the reading task, vowels, and words, as well as the average of all tasks, which we term global voice pitch. The median was used as it is more resistant to outliers than the mean. We represent voice pitch variability with *f*_o_ SD. We calculated *f*_o_ skew (asymmetry around the mean) as another measure of distribution in voice pitch. For vowel acoustics, we estimated formants *F*_1_, *F*_2_, *F*_3_, and *F*_4_ (median and SD) from the isolated vowel task. Last, on the basis of formant values in the vowel task, we computed three aggregate vowel measures: two vowel space metrics that are sensitive to dysarthria, namely, quadrilateral vowel space area [VSA4; (*34*)] and formant centralization ratio [FCR; (*35*)], and calculated apparent vocal tract length (VTL) based on formants *F*_1_ to *F*_4_ (*24*, *36*) (Materials and Methods). In total, we used 72 measures in the subsequent analysis (see table S1 for a list of the phenotypes and table S2 for mean values).

Not surprisingly, many of the measures are highly correlated (table S3 and figs. S1 and S2). For instance, voice pitch in reading is strongly correlated with voice pitch in vowels (males *r* = 0.81; females *r* = 0.70), words (males *r* = 0.80; females *r* = 0.70), and global voice pitch (males *r* = 0.85; females *r* = 0.77; all *P* < 1.0 × 10^−300^). Voice pitch is positively correlated with pitch variability (e.g., *f*_o_ median and *f*_o_ SD in reading: males *r =* 0.43, *P* = 2.7 × 10^−252^; females *r* = 0.14, *P* = 7.5 × 10^−35^). Correlations between vowel formants span a wide range, depending on the vowel and the formant. The strongest within-vowel correlation is between *F*_1_ and *F*_2_ in the vowel /ɔ/ (as in "walk;" males *r =* 0.62; females *r =* 0.75; both *P* < 1.0 × 10^−300^). Aggregated vowel metrics are correlated with voice pitch in reading, indicating slightly more vowel clarity with higher voice pitch (e.g., VSA4; males *r* = 0.13, *P* = 2.7 × 10^−24^; females *r* = 0.16, *P* = 2.0 × 10^−42^).

### Voice and vowel acoustics across the life span

**Fig. 1.**
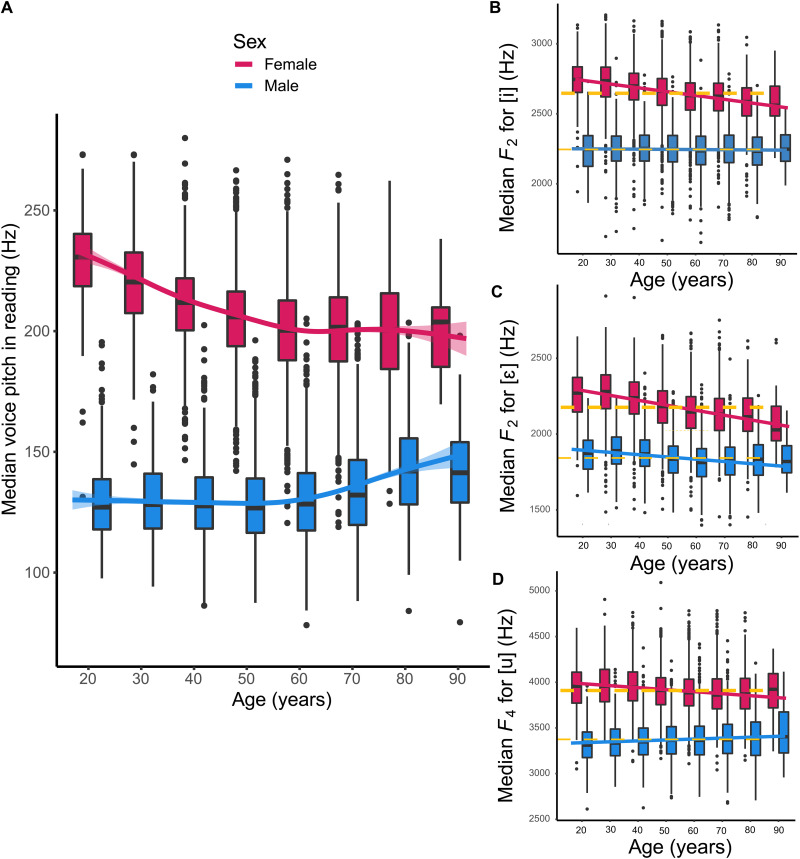
Voice pitch and vowel acoustics across the life span. (**A**) Voice pitch in reading (median *f*_o_ in hertz) is plotted for males and females according to age. For each age group and sex, the bottom and top of the boxes indicate the bottom and top quartiles, the line inside the box indicates the median, the whiskers indicate the most extreme values inside 1.5 times the interquartile range, and the dots indicate values outside that range. (**B**) Vowel formant *F*_2_ is plotted (in hertz) according to age and sex in the vowel [i] (similar to the vowel in English "meet"). (**C**) Vowel formant *F*_2_ in vowel [ɛ] ("met"). (**D**) Vowel formant *F*_4_ in vowel [u] ("boot").

The age range of participants was 18 to 93 years (mean age 54.1 years, SD 14.7, 56% females). Female voice pitch in reading decreases by 0.8 Hz per year [95% confidence interval (CI), 0.7 to 0.8] up until around 60 years of age, whereafter it remains stable: from 223.3 Hz at 20 to 30 years (95% CI, 221.6 to 230.2) to 197.4 Hz at the age of 60 (95% CI, 196.5 to 198.2) (Fig. 1A). Age-related changes in male voice pitch are quite different; voice pitch is stable at 124.0 Hz (95% CI, 123.4 to 124.6) up until around 60 years of age, whereafter it increases by 0.6 Hz per year (95% CI, 0.5 to 0.8) and reaches 136.5 Hz by the age of 90 (95% CI, 128.2 to 144.8). Formant values change differently with age between the sexes and for different vowels (Fig. 1, B to D; table S4; and figs. S1 to S4). Age effects are generally more frequent and pronounced in women. Most often, the age effects have a concordant direction in both sexes (Fig. 1C) but with some exceptions (Fig. 1D).

### Correlations with anthropometric, physiological, and cognitive traits

To test genetic association with these vocal traits, we inverse normal transformed the acoustic measures separately for each sex and adjusted for age, body mass index (BMI), and height, as voice and vowel measures are sexually dimorphic and influenced by body size (*24*). Because there is limited knowledge about the biological correlates of voice and vowel acoustics in large samples, we adopted a hypothesis-free approach and assessed the 72 adjusted voice and speech measures for correlation with more than 20,000 other phenotypes available for the participants of this study, including diagnostic codes, anthropometrics, blood measurements, cognitive tests, and various self-reports, using a Bonferroni significance threshold of 3.2 × 10^−8^ (0.05/72 × 21,885). We first performed this analysis on data for both sexes combined for increased power (table S5). We found numerous correlations, mainly with cognition and dual x-ray absorptiometry (DXA) body composition and other anthropometric measurements. A few findings are highlighted below, all of which show concordant effects in both sexes (table S5).

**Fig. 2.**
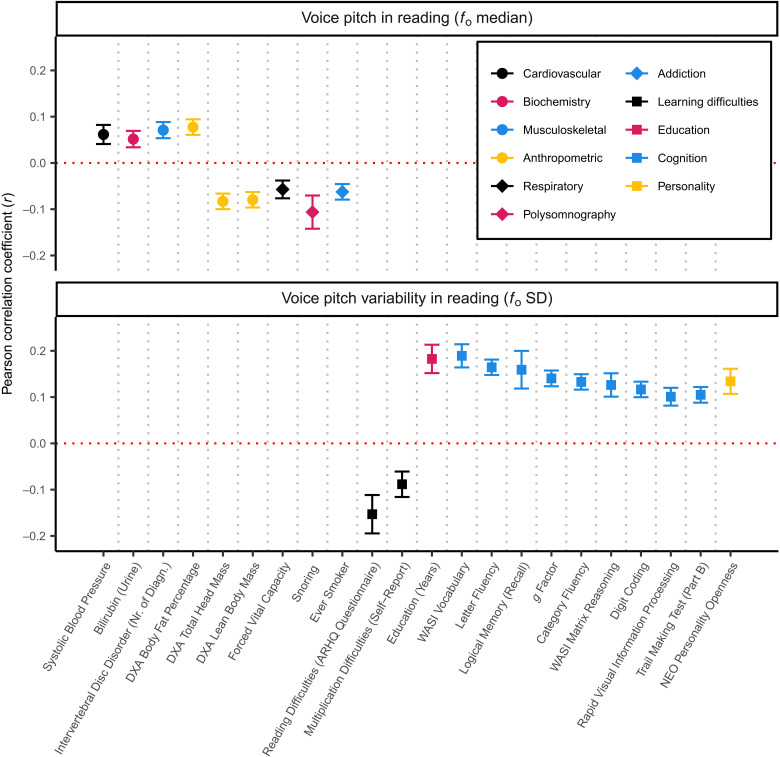
Phenotypic correlations with selected voice measures. Representative correlations (Bonferroni threshold 3.2 × 10^−8^) with other phenotypes for voice pitch in reading (*f*_o_ median) and voice pitch variability in reading (*f*_o_ SD) are shown. Each point with 95% CIs reflects the correlation with a phenotype, the *x* axis shows the phenotype, and the *y* axis displays the correlation coefficient (*r*). The direction of correlation for Trail Making Test (measured in seconds) was reversed to reflect better performance.

Voice pitch is mainly linked to anthropometric and physiological traits (Fig. 2 and table S5). For instance, higher median voice pitch in reading is correlated with reduced head mass (*r* = −0.08, *P* = 1.6 × 10^−20^), reduced lean body mass (*r* = −0.08, *P* = 6.0 × 10^−19^), and higher total body fat percentage (*r* = 0.08, *P* = 5.6 × 10^−18^). In addition, noteworthy are correlations between higher voice pitch in reading with negative health indicators, including higher systolic blood pressure (*r* = 0.06, *P* = 1.4 × 10^−8^), increased levels of bilirubin in urine (*r =* 0.05, *P* = 3.1 × 10^−8^), and reduced forced vital capacity, a measure of respiratory muscle function (*r* = −0.06, *P* = 1.6 × 10^−8^).

Voice pitch variability in reading (*f*_o_ SD; Fig. 2) correlates mainly with cognitive measures, including verbal fluency tasks (Letter Fluency: *r* = 0.17, *P* = 9.2 × 10^−76^, Category Fluency: *r* = 0.13, *P* = 7.6 × 10^−50^) and the *g* factor, a measure of general cognitive ability based on verbal fluency, executive functioning, and spatial working memory tasks (*37*) (*r* = 0.14, *P* = 1.8 × 10^−53^). Voice pitch variability in reading also correlates positively with the personality measure openness (*r* = 0.13, *P* = 2.1 × 10^−20^), in agreement with our prior findings linking verbal ability and openness (*37*). These correlations may simply reflect more lively intonation with increased verbal ability and hence ease of reading, as increased voice pitch variability also correlates with reduced reading difficulties reported on the Adult Reading History Questionnaire (*38*) (*r* = −0.15, *P* = 3.5 × 10^−12^). Effects of low literacy skills on pitch span have been observed in studies on bilinguals reading in their second language (*39*). Thus, whether these correlations hold for spontaneous speech is unclear.

Vowel formants correlate with both anthropometrics and cognition. For anthropometric traits, the strongest correlations are with vowel formants *F*_2_ and *F*_3_. For instance, *F*_2_ in [i] (as in "meet") correlates negatively with lumbar spine area measured with DXA (vertebrae L1 to L3 in the lower back; *r =* −0.11, *P* = 4.1 × 10^−28^) and lean trunk mass (*r* = −0.08, *P* = 9.5 × 10^−21^). In general, as with voice pitch, decreased *F*_2_ and *F*_3_ in the average of all vowels correlate with increased head mass, sitting height, and lean muscle mass, but decreased body fat (table S5). These findings are largely in line with prior reports that increased body size goes in hand with lower vowel formants (*24*), but additionally demonstrate that even when adjusting for height and BMI, formants are correlated with more fine-grained anthropometric measures such as fat versus lean body mass.

Correlations between vowel formants and cognition are mainly for *F*_1_, *F*_2_, and *F*_3_. The strongest is for *F*_3_ in [i], which is correlated positively with the *g* factor (*r* = 0.12, *P* = 1.1 × 10^−41^). Vowel formant frequencies extracted from words have been associated with variation in cognitive load (*40*), presumably because working memory requirements affect task performance including speech (*41*), but the mechanistic link between cognition and vowel formants in isolated vowels is unknown. A higher VSA4, which is indicative of clear (nondysarthric) speech (*34*), is correlated with better cognitive function, including a higher *g* factor (*r* = 0.07, *P* = 2.2 × 10^−15^), but reduced lean trunk mass (*r* = −0.05, *P* = 1.7 × 10^−9^).

Sex-specific analyses revealed largely similar results (table S5). Of 1021 associations significant in either or both sexes, 18 differed significantly between the sexes according to the Bonferroni-adjusted *P* value for heterogeneity in effect sizes (*P*_het_; threshold 4.9 × 10^−5^). Most of these were correlations between vowel formants and three-dimensional body scan measures (e.g., the correlation between *F*_2_ in the average of all vowels with the distance from waist to knee; *P*_het_ = 4.7 × 10^−8^; table S5). The results of this exploration may generate hypotheses that can be followed up in further studies.

### Heritability of voice and vowel acoustics

Using linkage disequilibrium (LD) score regression, we estimated the SNP-based heritability of the voice pitch and vowel measures (*h*^2^-SNP) (Materials and Methods) (*42*). *h*^2^-SNP provides an estimate of phenotypic variance explained by common sequence variants. Nonzero heritability for a particular phenotype indicates that it is likely to be in some sense well measured and influenced by DNA sequence variants. We found that voice pitch, voice pitch variability, and vowel formants have a small-to-modest *h*^2^-SNP, but the measures show some variability across vowels and elicitation tasks (table S6). Differences in *h*^2^-SNP between correlated phenotypes may reflect true differences in heritability or, alternatively, that the phenotypes differ in measurement accuracy (e.g., due to task demands or factors in the acoustic analysis; Supplementary Text). The highest *h*^2^-SNP for voice pitch was 17% (in reading; 95% CI, 9 to 24%), while for voice pitch variability, it was 13% (also for reading; 95% CI, 5 to 20%). Of the vowel measures, the highest *h*^2^-SNP was 14% for *F*_2_ (in average of all vowels; 95% CI, 7 to 21%), 9% for *F*_1_ (in vowel [a]; 95% CI, 2 to 15%), 8% for *F*_3_ (vowel [ɔ], as in "walk"; 95% CI, 1 to 15%), and 9% for *F*_4_ (vowel [i]; 95% CI, 1 to 17%). Of 72 measures, 41 have very low heritability (*h*^2^-SNP < 5%), including voice pitch skew and most of the measures for the SD of vowel formants.

### Genetic correlations

Using cross-trait LD score regression (*42*), we estimated the genetic correlation of the three most heritable phenotypes, i.e., voice pitch in reading, voice pitch variability in reading, and *F*_2_ in the average of all vowels, with summary statistics for 13 available meta-analyses and relevant traits in the UK Biobank, based on the phenotypic correlations and prior literature (table S7). These traits include, for instance, the *g* factor, total lean mass, systolic blood pressure, maximum grip strength, relative age voice broke, number of live births, and number of children fathered (table S7). Using a threshold based on Bonferroni correction (0.05/3 × 13 = 0.001), we found a positive genetic correlation between education (no. of years) in the UK Biobank and voice pitch variability in reading (*rg* = 0.30, *P* = 7.4 × 10^−6^), in line with the phenotypic correlations between voice pitch variability in reading and measures of cognition and verbal ability. We also found a negative genetic correlation between ever versus never smoking in the UK Biobank and voice pitch in reading (*rg* = −0.20, *P* = 0.0009), indicating that higher voice pitch is genetically correlated with never smoking.

### Variants in *ABCC9* associated with voice pitch

We tested the 72 adjusted voice and speech measures for association with genotypes using 39.2 million high-quality sequence variants, detected through whole-genome sequencing of 63,460 Icelanders and imputed into 173,025 chip-typed Icelanders and their relatives, including the 12,901 participants of this GWAS study (*43*, *44*). We tested for association under an additive model and considered associations significant if the *P* value was below a weighted genome-wide significance threshold based on variant annotation (*45*). Variants at three loci met the Bonferroni threshold (table S8). One locus passed further correction for the number of phenotypes tested.

**Fig. 3.**
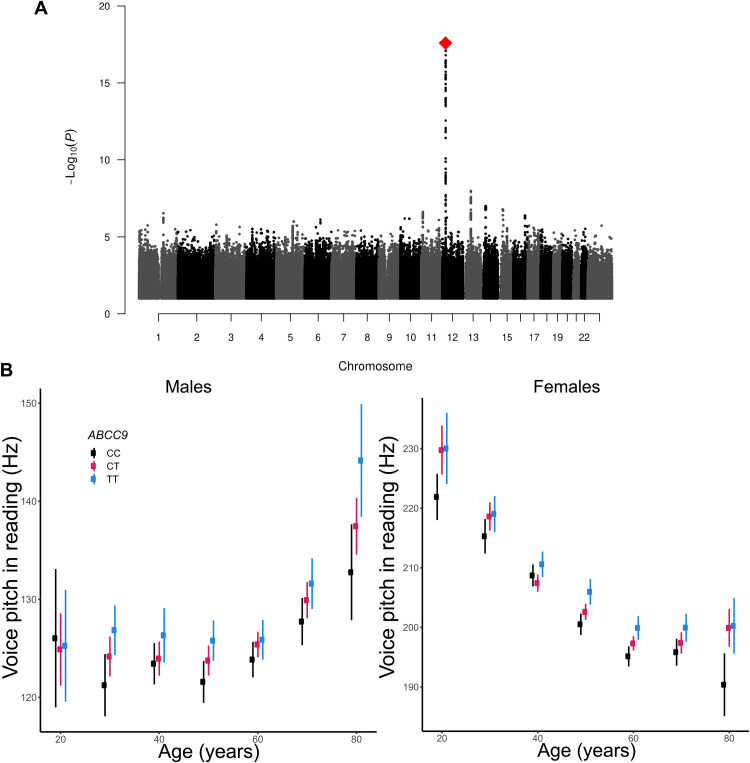
Association with voice pitch. (**A**) Manhattan plot of the association results for voice pitch in reading (median *f*_o_). Each variant in the genome is represented as a black or gray point and positioned according to its chromosomal position (*x* axis) and the −log_10_ *P* value for association with voice pitch in reading (*y* axis). rs11046212-T is indicated with a red diamond. (**B**) Voice pitch in reading, presented in hertz, as a function of rs11046212-T genotype and age in males and females.

A common intronic variant, rs11046212-T in adenosinetriphosphate (ATP) binding cassette subfamily C member 9 gene (*ABCC9*) on chromosome 12, is associated with higher voice pitch (*f*_o_ median) in the reading task (β = 0.11 SD, *P* = 2.6 × 10^−18^; Fig. 3). rs11046212-T shows a comparable association with voice pitch in reading when height and BMI are not used as covariates (β = 0.11 SD, *P* = 3.2 × 10^−17^). *ABCC9* encodes sulfonylurea receptor 2 (SUR2), which forms a regulatory subunit of ATP-sensitive potassium (K_ATP_) channels (*46*, *47*). The allele frequency (AF) of rs11046212-T in Iceland is 47.7%. The effect on voice pitch in reading (adjusted for age and sex) is 2.1 Hz per allele, a sizable effect particularly in homozygotes (4.2 Hz). rs11046212-T is also associated with a higher-pitched voice in words (β = 0.10 SD, *P* = 3.1 × 10^−15^), vowels (β = 0.10 SD, *P* = 1.6 × 10^−14^), and the global average of all speech tasks (β = 0.10 SD, *P* = 1.2 × 10^−14^). Because voice pitch is a sexually dimorphic trait (*17*, *48*), we also performed the association analysis for males and females separately, but the effect of rs11046212-T is not different between the sexes (*P*_het_ = 0.62).

### Association of *ABCC9* variants with gene expression and cardiovascular traits

**Fig. 4.**
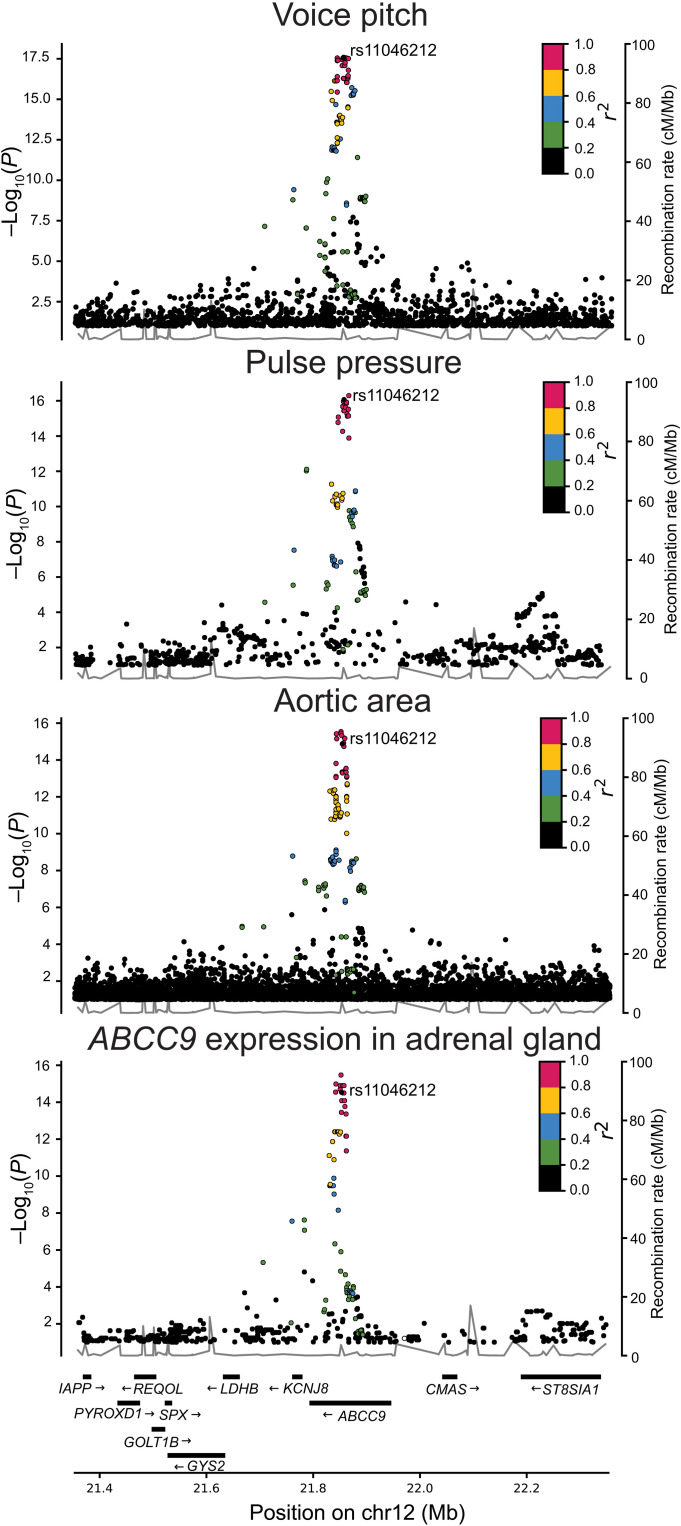
Regional plots of *ABCC9* variants associated with voice pitch, pulse pressure, aortic area, and *ABCC9* expression in adrenal gland. Variants are plotted according to their genomic position (*x* axis) and the −log_10_ *P* value for association with the phenotype (left *y* axis). The lead variant rs11046212-T is shown as purple; other variants are colored to reflect their correlation (LD) with the lead (those in red are highly correlated with the lead variant). Nearby genes are shown on the bottom.

Credible set analysis (*49*) identified 15 variants in *ABCC9* as probable causal variants for the voice pitch association signal, including 14 SNPs and one indel (correlation with lead *r*^2^ > 0.84; table S9). We performed colocalization analysis of correlated variants (*r*^2^ > 0.8) associated with gene expression and other GWAS traits. One of the variants in the credible set, *ABCC9* rs2307024-G (correlation with lead variant *r^2^* = 1), is a lead SNP associated with reduced *ABCC9* expression in adrenal gland (effect size = −0.64 SD, *P* = 3.4 × 10^−16^, GTEx v8). This variant (rs2307024-G) has also been reported as a top variant associated with reduced ascending aortic diameter (*50*) and is associated with reduced ascending aortic area in our cardiovascular magnetic resonance imaging (MRI) analyses of the same data source (β = −0.06 SD, *P* = 4.7 × 10^−16^; table S9). Another variant in the credible set, rs704191-T (*r^2^* = 0.85), has been reported as associated with greater pulse pressure (*51*, *52*) and is also associated with pulse pressure in our meta-analysis of more than 900,000 European individuals (β = 0.16 SD, *P* = 5.1 × 10^−17^; table S9). Thus, the *ABCC9* variants associated with higher voice pitch are also associated with greater pulse pressure, reduced ascending aortic area, and reduced expression of *ABCC9* in the adrenal gland (Fig. 4). We tested the lead variant rs11046212-T for association with other phenotypes in our dataset, including the cognitive and anthropometric traits described above, but found no additional associations accounting for the number of tests performed (*P* > 0.05/21,885 = 2.3 × 10^−6^).

### Fine-mapping in individuals of African ancestry

For 11 of the 14 SNPs in the credible set, the allele associated with higher voice pitch is derived (non-ancestral, 24 primates EPO-Extended, Ensembl) (table S9). The *ABCC9* voice pitch variants belong to a single class of LD in Europeans, with AF ranging from 38 to 42% (1000Genomes; table S9). The only populations that exhibit marked differences in AF for these variants, distributed over more than one LD class, have African ancestry (AF^Africans^, 5 to 29%; 1000Genomes; table S8). Hence, fine-mapping in Africans could help to further narrow down the set of credible variants. Although we could not obtain voice pitch GWAS or adrenal gene expression results for Africans [expression quantitative trait loci (QTL) are analyzed from GTEx, which is mainly, but not exclusively, composed of European individuals (*53*)], we did have access to GWAS results for pulse pressure, tested among individuals of African ancestry in the UK Biobank (*54*). We assessed the credible set of 15 variants for association with pulse pressure in people of African ancestry [UK Biobank (*54*), *N* = 9482]. Only four of these 15 variants, belonging to two LD classes in Africans (AF^Africans^ ca. 5% versus 15%), showed a nominal association with pulse pressure in individuals of African ancestry (rs10841900-A, rs11046213-T, rs861202-G, and rs1643235-C; table S8). All four alleles associated with higher pitch are derived. The available data suggest these four to be reasonable candidates for being the causal variant behind the pulse pressure association. We note that the frequency of these four derived higher-pitch alleles varies considerably between populations, from 5 to 16% in Africans to 38 to 40% in Europeans and 49 to 50% in South Asians.

### Fixed differences between humans and primates in *ABCC9*

Given that humans are unique among the great apes in their extensive use of vocalizations, we were interested in whether *ABCC9* contains fixed human-specific alleles that could have a more fundamental role in the common genetic architecture underlying the human voice. We used a multispecies alignment of primate reference genomes (13 primates EPO, Ensembl r100) and databases of human sequence variants [UK Biobank (*54*) and 1000Genomes phase 3 release V5] to seek fixed single-nucleotide changes in *ABCC9* (Materials and Methods). Fixed changes in humans were defined as sites where chimpanzee, bonobo, and rhesus macaque all carry the ancestral allele, while *Homo sapiens* carries a derived allele at a frequency of more than 99.99%. Of 17,967 protein coding genes, 49.3% have one or more fixed change in the coding or splice region according to these criteria. We identified one fixed change in *ABCC9* predicted to be a missense variant (table S10). The fixed change in *ABCC9* (p.Asn1538Asp, chr12:21801082) is located in the last coding exon of *ABCC9* transcript variant *SUR2B* (NM_020297.3). *SUR2B* and *SUR2A* are two main mRNA transcripts produced by differential splicing of *ABCC9* and differ only in the alternative last coding exons (*55*, *56*). Humans have T at chr12:21801082, resulting in an asparagine (Asn) amino acid at codon 1538, while all other primate genomes available in Ensembl carry the ancestral C nucleotide, resulting in aspartate (Asp) (24 primates EPO-Extended, Ensembl). We examined four genomes, sequenced to high depth, from archaic humans, i.e., the Vindija (*57*), Altai (*58*) and Chagyrskaya (*59*) Neanderthals, and the Denisovan (*60*), and determined that all four are homozygotes for the allele that is fixed in modern humans (T; table S10). Thus, this change occurred after hominins split from the great apes but before they diverged into modern humans, Neanderthals, and Denisovans.

p.Asn1538Asp is located in the ABC transporter domain, close to the ATP binding side. Aspartate can be negatively charged and hence can form salt bridges with nearby amino acids that are positively charged. In contrast, the fixed asparagine in humans is uncharged and cannot form salt bridges. In general, *ABCC9* is relatively intolerant to inactivation, with a loss-of-function observed/expected upper bound fraction (LOEUF) (*61*) of 0.482 (GnomAD), compared to an average score of 0.916 for all protein coding genes in our analysis. The ratio of nonsynonymous to synonymous substitutions (dN/dS) in *ABCC9* is 0.126 [GenEvo (*62*)], indicating that the gene is constrained (values of <1 indicate negative selection). The combined annotation-dependent depletion score (CADD) for the primate C nucleotide is 19.01 (PHRED); a score of 20 indicates that a variant is among the top 1% of deleterious variants in the human genome. We searched for variation at chr12:21801082 in the largest available databases of sequenced genotypes—UK Biobank (*54*) (150,119 genomes), TOPMed Freeze 8 (132,345), and GnomAD v3.1 (76,156)—and found no individuals carrying a SNP at this location. None of these results gives reason to suppose that the fixed p.Asn1538Asp variant had a major functional impact. However, they also do not rule out this possibility. In particular, the lack of individuals with alternative alleles at chr12:21801082 in large sequence databases at least provides some grounds for further investigation.

## DISCUSSION

Using the largest sample of speech recordings with corresponding genotypes to date, we have taken the first steps toward understanding the genetic basis of variation in the human voice and speech. We discovered sequence variants in *ABCC9* that associate with voice pitch, irrespective of sex. rs11046212-T and correlated common intronic variants in *ABCC9* are associated with a higher-pitched voice, greater pulse pressure [a marker of cardiovascular risk (*63*)], reduced ascending aortic area, and reduced expression of *ABCC9* in the adrenal gland.

The *ABCC9* protein SUR2 forms a regulatory subunit of K_ATP_ channels (*46*). K_ATP_ channels act as brakes on excitability in multiple tissues, regulating cellular functions such as hormone secretion, vascular tone, cardiac muscle contraction, and synaptic transmission (*47*, *64*). Hitherto, *ABCC9* has not been directly linked to voice or speech phenotypes. However, very rare gain-of-function mutations in *ABCC9* under a dominant mode of inheritance cause Cantú syndrome, a disorder characterized by excess hair growth (hypertrichosis) as well as cardiovascular, craniofacial, and skeletal abnormalities and, in some cases, mild speech delays and hoarse voice (*65*–*68*). Moreover, *ABCC9* mutations have been associated with dilated cardiomyopathy and atrial fibrillation (*69*, *70*). Biallelic loss-of-function mutations in *ABCC9* are linked to intellectual disability myopathy syndrome (AIMS), with neurological symptoms such as mild-to-moderate cognitive impairments, white matter abnormalities, and anxiety (*71*).

The mechanism of *ABCC9* action on voice pitch is not yet clear. The adrenal glands, where rs11046212-T associates with *ABCC9* expression, produce several steroids known to influence voice pitch. These include cortisol and adrenal androgens such as dehydroepiandrosterone (DHEA) and DHEA sulfate (*17*, *72*, *73*), which are precursors to testosterone and other sex hormones. The association of voice pitch variants with pulse pressure and aortic area may also implicate nonhormonal mechanisms. Greater pulse pressure is tightly linked to reduced aortic diameter and increased vascular stiffness, the latter of which is mainly determined by the content and properties of elastin and collagen in the layers of the arterial wall (*74*, *75*). Both elastin and collagen are also critical proteins in the vocal folds, contributing to the biomechanics that allow for vocal fold vibration (*76*, *77*). The hair loss drug minoxidil (Rogaine), which is a K_ATP_ channel agonist that binds to SUR2B (*65*), has been shown to increase elastin synthesis and elastin gene transcription (*78*). An alternative is that *ABCC9* variants act on voice pitch through the vocalis muscle in the vocal folds or muscle in the vocal tract, given the high expression of SUR2 proteins in skeletal and smooth muscle (*56*). Further research could clarify the pathways through which *ABCC9* variants influence the human voice.

The *ABCC9* variants we propose as causal candidates, based on fine-mapping of the variants in individuals of African ancestry, have in common that the derived alleles are associated with higher voice pitch. A GWAS for voice pitch in African populations (or replication tests for loci in the *ABCC9* region) could test this hypothesis.

Under the assumption that sequence variants associated with normal variation in voice and speech may be located in genes that also play a more fundamental role in the genetic architecture of the human vocal system, we searched for fixed differences between humans and nonspeaking primates in *ABCC9.* We noted a missense change in *ABCC9* (p.Asn1538Asp, chr12:21801082) that is fixed in humans but not present in primate reference genomes, located in the *ABCC9* transcript variant *SUR2B*. *SUR2B* shows abundant expression in smooth (nonvoluntary) muscles and the brain (*55*, *56*). About half of protein coding genes have one or more fixed change of moderate or high impact according to our analysis, and thus, the importance of this fixed change is uncertain. However, due to the different properties of the two amino acids that are exchanged (aspartate for asparagine) and their location, it is a possibility that p.Asn1538Asp alters the structure of the ABC transporter domain. *ABCC9* is otherwise evolutionarily constrained. There appear to be no known human carriers of an alternative allele at this position in large sequence databases (UK Biobank, TOPMed Freeze 8, and GnomAD v3.1). Thus, while it is not possible to state that this fixed change has some impact, it clearly warrants further consideration in relation to the voice and other human traits.

Our findings indicate that voice pitch, voice pitch variability, and vowel formants have a small-to-modest heritable component, with *h*^2^-SNP of a magnitude similar to that of traits such as major depression (*h*^2^-SNP = 9%) (*79*) and personality (*h*^2^-SNP = 9 to 18%) (*80*). Our findings also show that these acoustic measures correlate with anthropometric, physiological, and cognitive traits. In particular, higher voice pitch is correlated with reduced lean body mass, an important determinant of bodily strength, as well as negative health metrics including higher systolic blood pressure. Moreover, we found that voice pitch variability in reading is linked to cognition, while vowel formants from isolated vowels correlate with both cognition and anthropometric traits. We note that these exploratory correlations, although statistically significant after multiple testing correction, are quite small.

Our study has a few limitations, particularly in relation to vowel formants. Errors in formant estimation may affect some vowel measures, as automatic vowel formant estimation is known to be error prone (Supplementary Text) (*81*). Lower *h*^2^-SNP for some vowels may therefore reflect difficulties in formant estimation rather than true differences in heritability. A seated posture may have a minor impact on the speech samples, particularly vowel formants, due to the tight connection between vocal tract position and formant frequencies. Vowel formants were extracted from isolated vowels, not running speech or individual words. More complex speech tasks, such as monologue, are more sensitive to articulation problems due to neurological factors as in Parkinson’s disease (*25*). Last, future studies should include measures of voice quality for additional insights into the neural and anatomical factors affecting the voice.

Speaking is one of the defining traits of our species, and yet, we know surprisingly little about the genetic components of voice and speech in humans. While hundreds of loci influencing intelligence and other key human traits have been discovered (*82*), the search for genome determinants of the vocal system—and linguistic abilities more generally—is still in its infancy (*83*), in part due to a lack of large genotyped cohorts with the relevant phenotypes. We hope that this study will be the first of many to examine genetic variation affecting the human voice and speech.

## MATERIALS AND METHODS

### Study population

Speech samples were obtained from 14,144 Icelandic participants as a part of the deCODE Health Study in Iceland, which involves comprehensive phenotyping of a general population sample. All participants were native speakers of Icelandic. Subjects donated a blood sample, were administered several physiological and neuropsychological tasks, answered health and lifestyle questionnaires, and authorized access to data from health registries and medical records. The study was approved by the National Bioethics Committee and the Data Protection Authority in Iceland (VSNb2015120006/03.01 with amendments). Personal identifiers were encrypted by a third-party system overseen by the Icelandic Data Protection Authority. All participants signed informed consent according to the Declaration of Helsinki.

### Recording

Speakers were seated in a sound-attenuated booth (350 Maxi Sound Shelter, IAC Acoustics) in front of a computer screen. Recordings were made with a professional DPA 4066-F omnidirectional condenser headset (70.2% of participants), an AKG HSC-271 cardioid condenser microphone headset (12.4%), or Aston Origin cardioid condenser studio microphone (18.3%). We changed microphones (from the studio microphone to head-mounted AKG HSC-271 and finally DPA 4066-F) to ensure standardized distance from the microphone and reduce noise in the recordings. The voice signals were sampled at 44.1 kHz with 16-bit resolution.

### Speech elicitation tasks

Custom computer software was used for elicitation of speech samples. Instructions were presented on the screen.

#### Read speech

Participants were first instructed to read a short story in Icelandic (183 words) from a handout at a comfortable pace and informed that their reading time was recorded (see table S10). The story concerns young kids sledding in snow. A longer version of the text was previously used in a study on reading ability. The text is relatively formal and contains several low-frequency words.

#### Isolated vowels

When prompted on the computer screen, participants produced two rounds of the Icelandic vowels commonly represented in the International Phonetic Alphabet (IPA) as [i] (similar to the English vowel in "meet"), [ɛ] ("met"), [a] ("ahead"), [ɔ] ("walk"), and [u] ("boot"), corresponding to í, e, a, o, ú in Icelandic orthography. [i, a, ɔ, u] are the corner vowels of Icelandic, i.e., most extreme in terms of tongue position (high-low or front-back) and corresponding formant values (*F*_1_ and *F*_2_) (*84*, *85*). Each vowel was presented on the computer screen using conventional orthography along with an example of a word containing the sound (Icelandic *ís *“ice cream,” ef “if,” amma “grandmother,” ofar “above,” úr “watch”). This task was preceded by a short practice.

#### Sustained [a]

Participants produced one sustained phonation of the vowel [a] for a duration of about 4 s. The sustained [a] was elicited without practice and involved a visual prompt (percentage of time completed and a corresponding circle filling up).

#### Words

Participants produced two rounds of two short words, initially chosen to probe lisp (rás “channel” and spila “play”).

### Acoustic analysis

#### Automatic speech recognition system for forced alignment

Automatic speech recognition and forced alignment were used as an automatic quality control, as utterances with very low acoustic likelihood score (reflecting mismatch between expected speech and the actual speech production) are removed, including outliers resulting from very poor speech quality or incorrect responses in the elicitation tasks. For this purpose, we used a triphone acoustic model trained on 542 hours of speech data, which was initially designed to transcribe Icelandic parliamentary speeches (*86*). The automatic speech recognition system was also used to remove silences in the recordings, including pauses between words in the reading.

#### Voice pitch

Fundamental frequency (*f*_o_) was estimated using Praat’s autocorrelation method, with a sex-specific setting (60 to 220 Hz for males, 100 to 300 Hz for females) (*87*). Over the duration of the whole vowel segment, an *f*_o_ contour was estimated from a sliding window analysis (with 60/40-ms-long windows for males/females and a 10-ms overlap). The resulting contour is further refined as the missing values are linearly interpolated and smoothed using median filtering over five neighboring values, and finally outlier values are automatically removed using the median absolute deviation (MAD) method. The reported values (median *f*_o_, SD *f*_o_, skew *f*_o_) are then estimated for each recording in Octave.

#### Vowel measures

Formant frequencies *F*_1_, *F*_2_, *F*_3_, and *F*_4_ were estimated from each short vowel recording with Praat’s “To Formant (burg)” formant frequency estimator. Here, the estimator was configured to extract a fifth formant frequency in addition to the four, with a sex-specific setting of the maximum formant frequency parameter (5000 Hz for male speakers, 5500 Hz for female speakers). Other common parameter settings used for this estimation include a time step of 0.01 s, a window length of 0.025 s, and a pre-emphasis applied in Praat using the default setting of 50 Hz. From the results generated by Praat, formant frequencies were extracted only at the time positions where the signal intensity was higher than 0.5 times the maximum intensity of the utterance, i.e., time positions with a relatively high voice intensity. The median and SD were calculated (median *F*_1_, median *F*_2_, median *F*_3_, median *F*_4_, SD *F*_1_, SD *F*_2_, SD *F*_3_, SD *F*_4_).

#### Aggregated vowel measures

Two measures were used to describe the vowel space spanned by formant frequencies of the vowels in the vowel task. The quadrilateral vowel space area (*34*) was calculated on the basis of *F*_1_ and *F*_2_ of the corner vowels [i, a, ɔ, u], with a polygonal area calculated from the four two-dimensional formant frequency vectors. Another vowel space measure is formant centralization ratio (*35*), which is defined by a formula that depends on *F*_1_ and *F*_2_ of the vowels [i, a, u]. Last, we estimated apparent VTL in centimeters using the VTL(deltaF) formula based on formant spacing (*24*, *36*), averaged for all short vowels ([i, ɛ, a, ɔ, u]), with estimations of formants *F*_1_, *F*_2_, *F*_3_, and *F*_4_ and *c* = 353 m/s for speed of sound.

### Quality control of speech samples

Of the 14,144 available speech samples for Icelanders, 32 were manually tagged as bad based on the distribution of the measures (0.2%). For the remaining samples, we required that at least 70% of the measures of interest could be estimated. A total of 1211 (8.6%) did not satisfy this criterion, in part because of technological issues with the recording, leaving 12,901 samples that were included in the subsequent analysis.

### Normalization and aggregate measures

We adjusted the logarithm of the acoustic measures separately for each sex for age, BMI, height, and microphone (*88*). For the repeated measures (vowels), we took the logarithm of the mean value. We then created aggregate vowel measures by computing the mean value of both tokens of each vowel (e.g., both recordings of [i]). We also created aggregate voice and vowel measures for the words and vowel tasks based on the mean of individual recordings. We applied rank-based inverse normal transformation to the adjusted measures before association testing. In total, 72 measures were submitted to association analysis.

### Genotype dataset

We analyzed 39.2 million high-quality sequence variants, detected through whole-genome sequencing of 63,460 Icelanders and imputed into 173,025 chip-typed Icelanders and their relatives (*43*, *44*). Whole-genome sequencing was performed using Illumina technology to a mean depth of at least 17.8× and a median depth of 36.9×. The sequence variants were jointly called using Graphtyper (*89*). We genotyped 173,025 Icelanders using Illumina SNP chips, and their genotypes were phased using long-range phasing (*90*). Genotypes of the 39.2 million sequence variants were imputed into all chip-typed Icelanders as well as relatives of the chip-typed, to increase the sample size for association analysis. All variants tested had imputation information over 0.95. Chromosomal positions are expressed according to GRCh38 (Genome Reference Consortium Human Build 38).

### Phenotypic correlations

We assessed the adjusted voice and speech measures for correlation with other traits available for the participants of this study using a Bonferroni significance threshold of 3.2 × 10^−8^ (0.05/72 × 21,885). These phenotypes were collected through the deCODE Health Study and other research projects at deCODE genetics. They include diagnostic codes, blood measurements, anthropometrics, cognitive tests, brain imaging, and various other traits, including self-reports. We only included correlations for which the sample size intersection of both traits assessed was over 100. For case-control studies, all individuals that were not listed as cases were treated as controls, and we further required at least one case and one control in the intersection of the two phenotypes. DXA body composition was measured in the deCODE Health Study using DEXA by Hologic (S/N200547). Cognition was tested with validated tasks such as Letter and Category Fluency, Spatial Working Memory, Logical Memory, Trail Making Test, Digit Coding, and Rapid Visual Information Processing (*37*), in addition to Matrix Reasoning and Vocabulary subtests of the Wechsler Abbreviated Scale of Intelligence.

### Heritability and genetic correlation

SNP-based heritability (observed scale) and genetic correlation were estimated using LD score regression (*42*). In these analyses, we used results for about 1.2 million well-imputed variants, and for LD information, we used precomputed LD scores for European populations (downloaded from: https://alkesgroup.broadinstitute.org/LDSCORE/). Genetic correlations with summary statistics were based on GWASs performed at deCODE Genetics using the UK Biobank resource, application number 56270.

### Association analysis

For each sequence variant, a linear regression model was used to test for association, using the genotype as an additive covariate, the transformed quantitative trait as a response, and assuming the variance-covariance matrix to be proportional to the kinship matrix (*43*). Associations were considered genome-wide significant if the *P* value was below a weighted genome-wide significance threshold based on variant annotation (*45*). The thresholds were estimated from the Icelandic data and corrected for multiple testing with a weighted Bonferroni adjustment using the enrichment of variant classes with predicted functional impact among association signals as weights. The weights were rescaled to control the family-wise error rate, resulting in significance thresholds of 2.5 × 10^−7^ for loss-of-function variants, 5.0 × 10^−8^ for moderate-impact variants, 4.5 × 10^−9^ for low-impact variants, 2.3 × 10^−9^ for other variants within DNase hypersensitivity sites, and 7.5 × 10^−10^ for remaining variants. Variants with quality flags (e.g., indicating issues with sequence variant calling or Hardy-Weinberg disequilibrium) were removed from analysis.

### Colocalization with eQTL, pQTL, and GWAS variants

We tested the lead variant for colocalization with correlated known variants (*r*^2^ > 0.8) in eQTL and pQTL datasets (based on 1-MB regions). eQTL datasets include both Icelandic data (not publicly available) and publicly available data, e.g., from the Genotype-Tissue Expression Consortium (GTEx v8). The pQTL data are based on plasma samples collected from 40,004 Icelanders measured using the SOMAscan platform. Last, we tested the lead variant for colocalization with known GWAS associations, based on the NHGRI-EBI GWAS Catalog (*91*) downloaded on 16 August 2021. We also obtained pulse pressure estimates by meta-analyzing data from Iceland (*N* = 149,988) and a recent GWAS based on data from the UK Biobank and the International Consortium of Blood Pressure–Genome Wide Association Studies (*51*) (*N* = 757,601). The Icelandic pulse pressure data were obtained from Landspitali—The National University Hospital of Iceland, the Primary Health Care of the Capital Area, as well as several deCODE studies (reference numbers VSNb2016020022/03.01, VSNb2015010033/03.12, VSNb2015030022/03.01, and VSNb2015030024/03.01). Ascending aortic area GWAS was based on the UK Biobank cardiovascular MRI data analyzed at deCODE Genetics (*N* = 33,150), using a spatiotemporal neural network to calculate the minimum and maximum cross-sectional ascending and descending aortic areas (*92*). The measurements extracted from the cardiovascular MR images were rank-transformed, inverse-normalized, and adjusted for age, age^2^, sex, sex × age, MR scanner type, and the first 10 principal components. The aorta analysis was approved through UK Biobank application number 56270.

### Credible set analysis

For the association signal, we created a credible set of SNPs that were 95% likely, based on posterior probability, to contain the causal disease-associated SNPs (*49*).

### Search for fixed derived variants

To seek fixed derived (non-ancestral) variants in *ABCC9,* we used a multispecies alignment of primate reference genomes (13 primates EPO, Ensembl r100). MafFilter (*93*) and GORpipe (*94*) tools were used for genome alignment and genotype calling. We defined human fixed derived variants as sites where *Pan paniscus*, *Pan troglodytes*, and *Macaca mulatta* carry the ancestral allele, while *H. sapiens* carries a derived allele with a frequency of more than 99.99% in databases of human sequence variants [UK Biobank (*54*) and 1000Genomes phase 3 release V5]. We searched for human fixed derived variants of high and moderate impact, based on VEP annotation (*95*).

### Comparison with archaic humans

For the credible voice pitch variants and the fixed missense change, we determined the alleles in four high-depth genomes from archaic humans, i.e., the Vindija (*57*), Altai (*58*) and Chagyrskaya (*59*) Neanderthals, and the Denisovan (*60*).

## Supplementary Materials

### This PDF file includes:

Figs. S1 to S6 Supplementary Text Legends for tables S1 to S11 References

## Notes

View/request a protocol for this paper from *Bio-protocol*.
