# Supplementary material for: Sequence variants affecting voice pitch in humans
Source: Sci Adv. 2023 Jun 9;9(23):eabq2969. doi: 10.1126/sciadv.abq2969 (PMC10256171; doi:10.1126/sciadv.abq2969)
Supplement: Supplementary file 1 — Figs. S1 to S6 Supplementary Text Legends for tables S1 to S11 References [file sciadv.abq2969_sm.pdf]

Science Advances
AAAS

Supplementary Materials for

Sequence variants affecting voice pitch in humans

Rosa S. Gisladottir *et al.*

Corresponding author: Email: Rosa S. Gisladottir, rosa.gisladottir@decode.is

Sci. Adv. **9**, eabq2969 (2023)
DOI: 10.1126/sciadv.abq2969

The PDF file includes:

Figs. S1 to S6
Supplementary Text
Legends for tables S1 to S11
References

Other Supplementary Material for this manuscript includes the following:

Tables S1 to S11

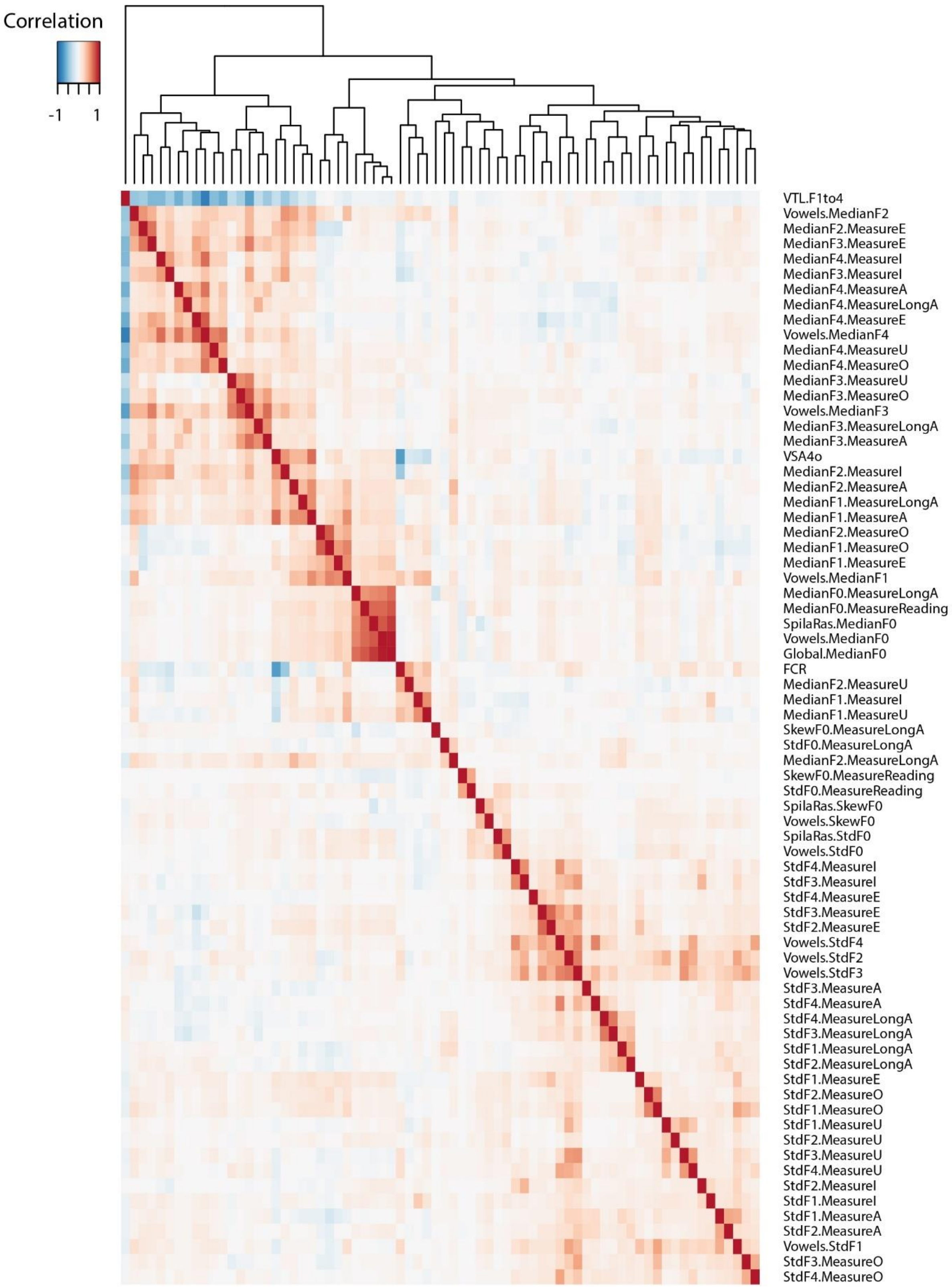

Fig. S1. Correlations between pairs of acoustic measures in females. The measures are ordered by the complete linkage hierarchical cluster method and the top of each panel shows the resulting dendrogram.

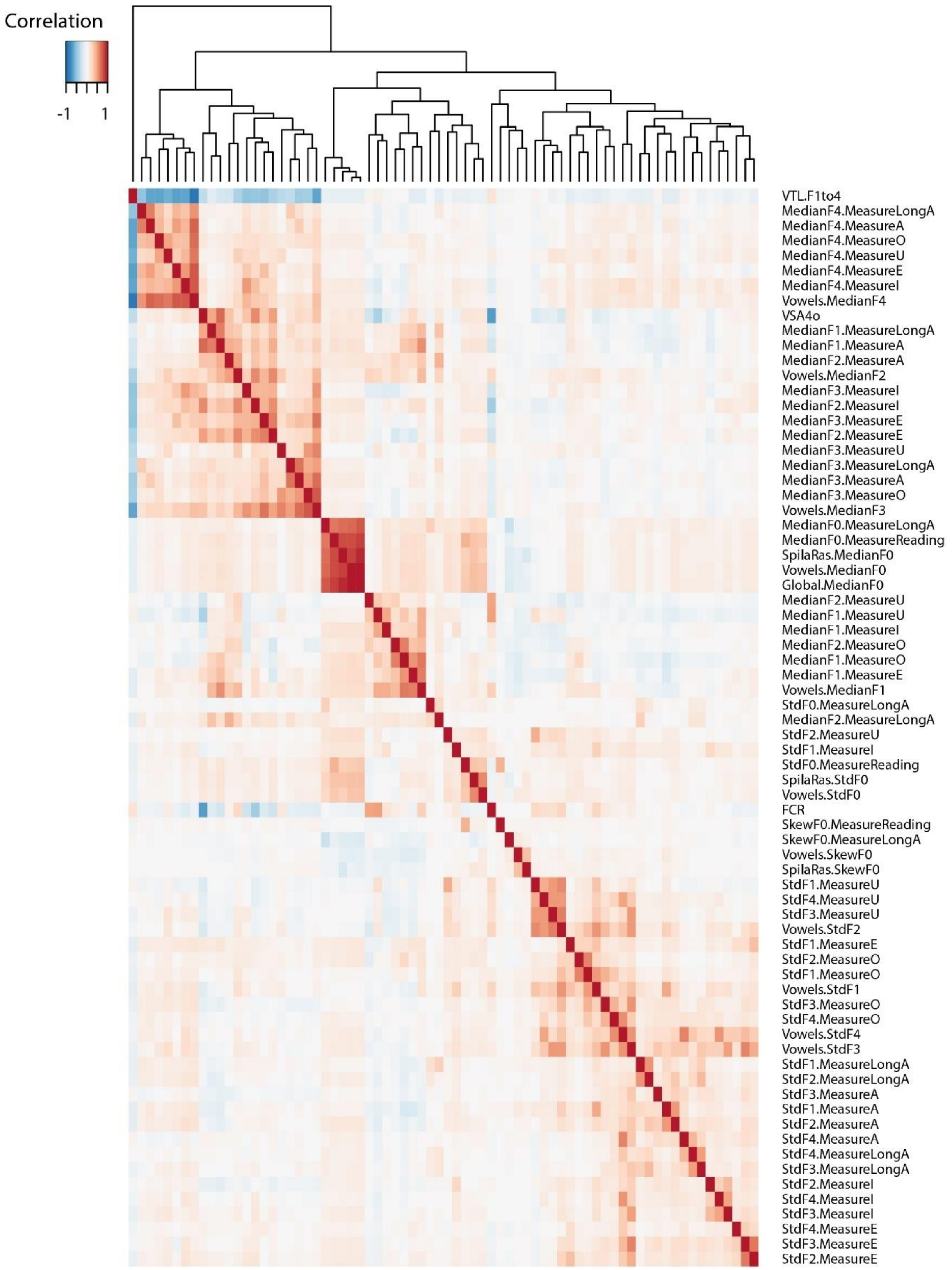

Fig. S2. Correlations between pairs of acoustic measures in males. The measures are ordered by the complete linkage hierarchical cluster method and the top of each panel shows the resulting dendrogram.

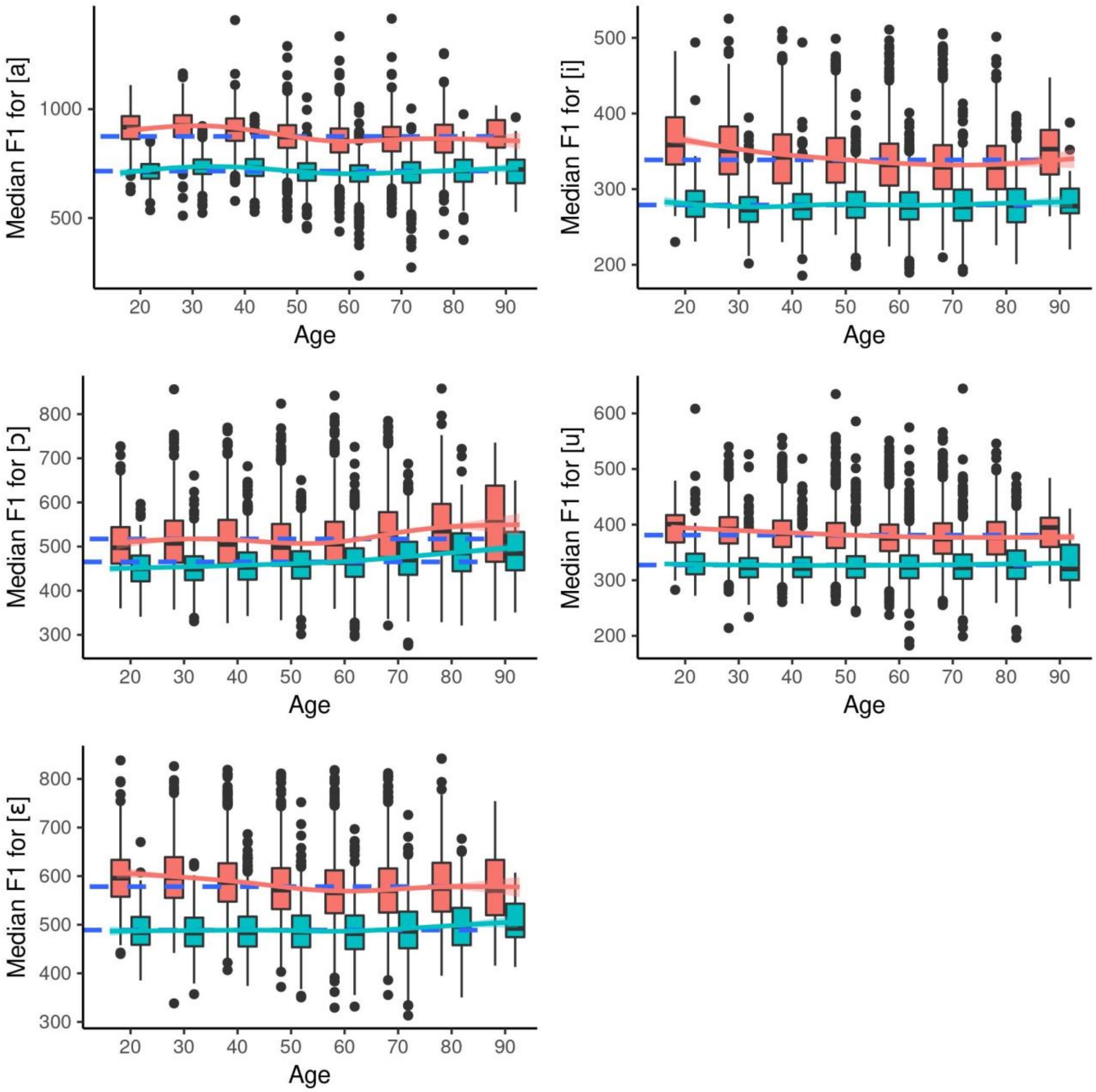

Fig. S3. Vowel formant 1 (F_1) across the lifespan. F_1 (in Hz) is plotted according to age (in years) and sex in the vowels [a], [i], [ɔ], [u] and [ɛ]. Blue boxes indicate values for males, red boxes values for females. For each age group and sex, the bottom and tops of the boxes indicate the bottom and top quartiles, the line inside the box indicates the median, the whiskers indicate the most extreme values inside 1.5 times the interquartile range, and the dots indicate values outside that range.

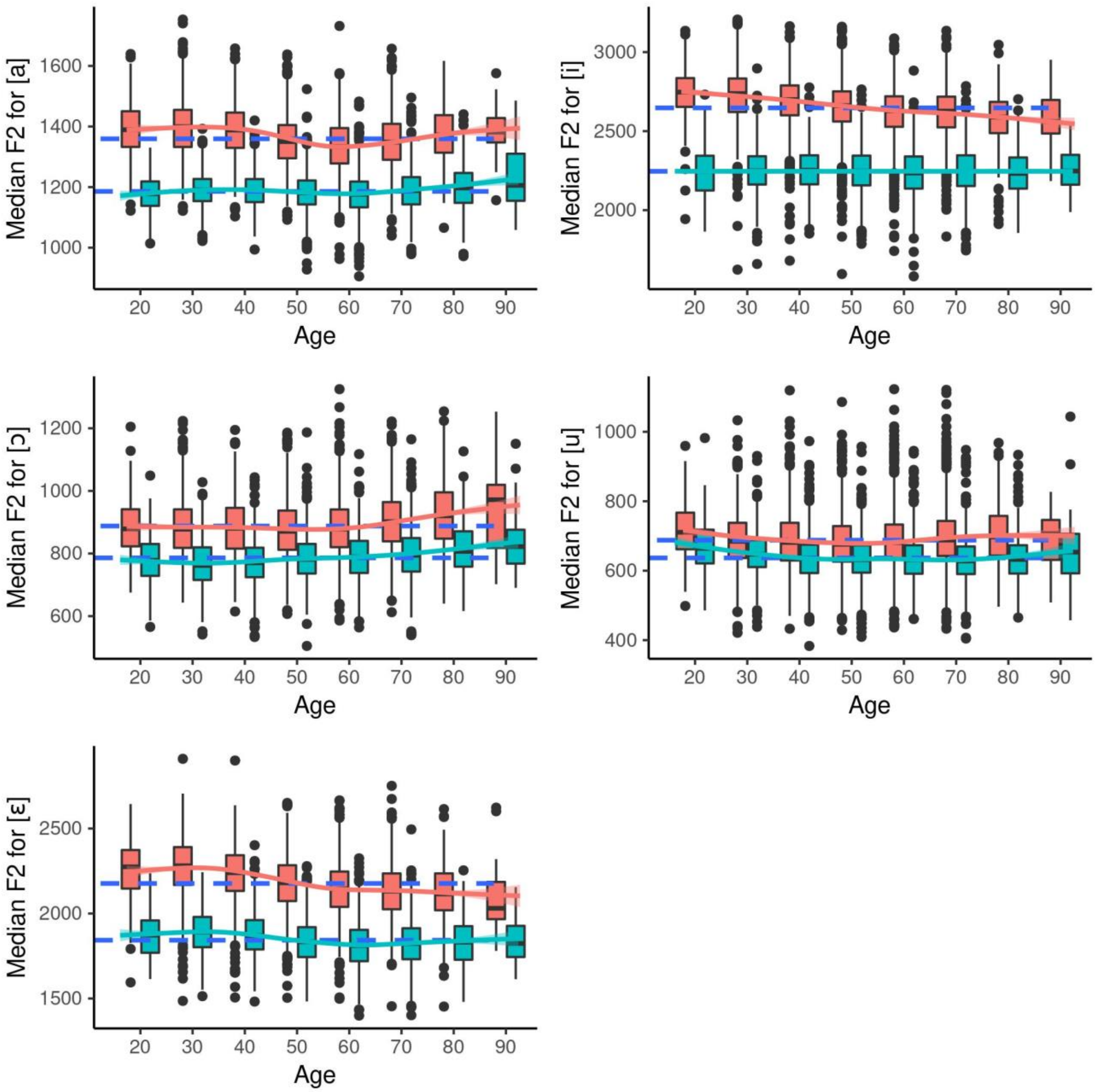

Fig. S4. Vowel formant 2 (*F*₂) across the lifespan. *F*₂ (in Hz) is plotted according to age (in years) and sex in the vowels [a], [i], [ɔ], [u] and [ɛ]. Blue boxes indicate values for males, red boxes values for females. For each age group and sex, the bottom and tops of the boxes indicate the bottom and top quartiles, the line inside the box indicates the median, the whiskers indicate the most extreme values inside 1.5 times the interquartile range, and the dots indicate values outside that range.

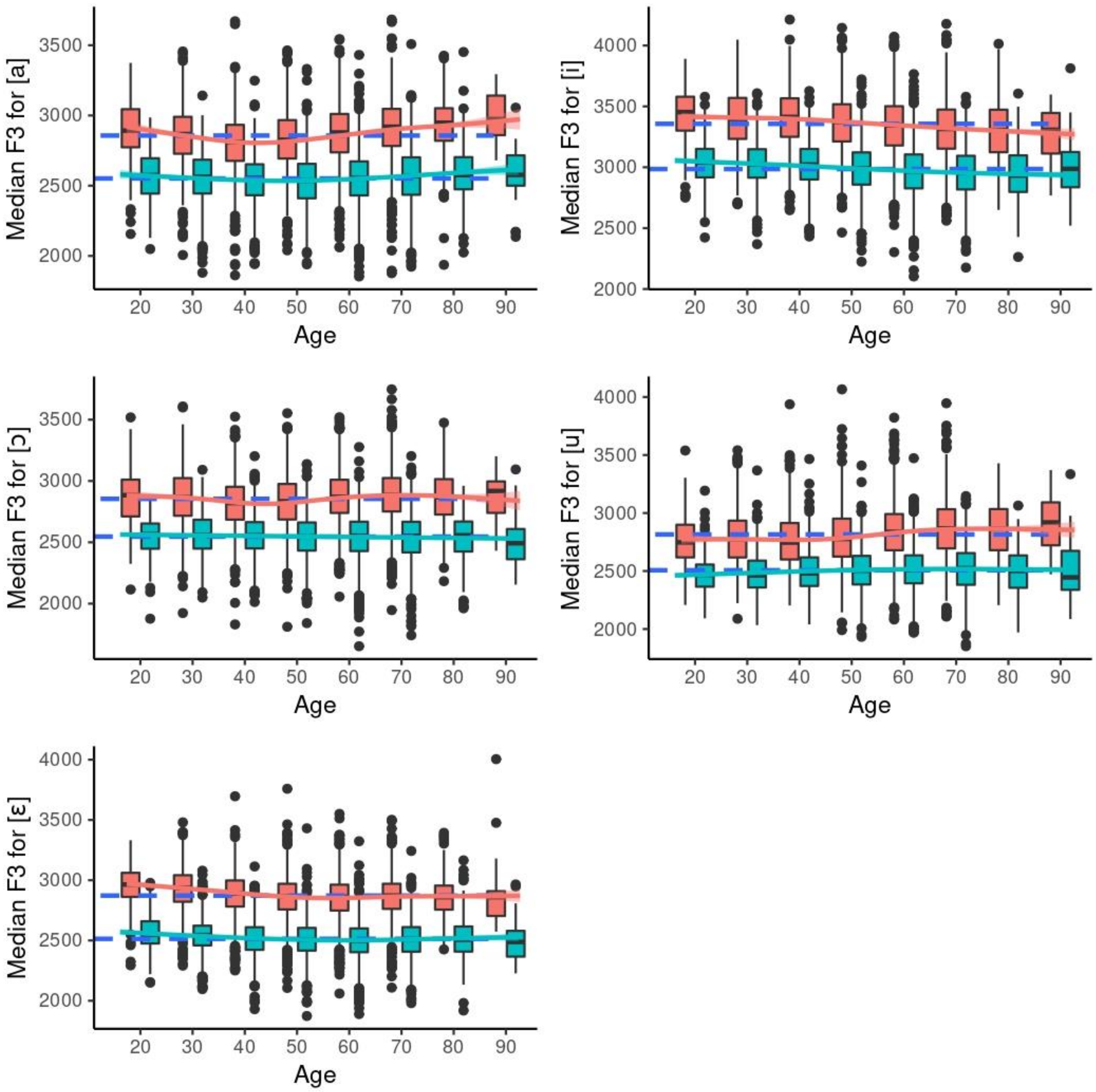

Fig. S5. Vowel formant 3 (F_3) across the lifespan. F_3 (in Hz) is plotted according to age (in years) and sex in the vowels [a], [i], [ɔ], [u] and [ɛ]. Blue boxes indicate values for males, red boxes values for females. For each age group and sex, the bottom and tops of the boxes indicate the bottom and top quartiles, the line inside the box indicates the median, the whiskers indicate the most extreme values inside 1.5 times the interquartile range, and the dots indicate values outside that range.

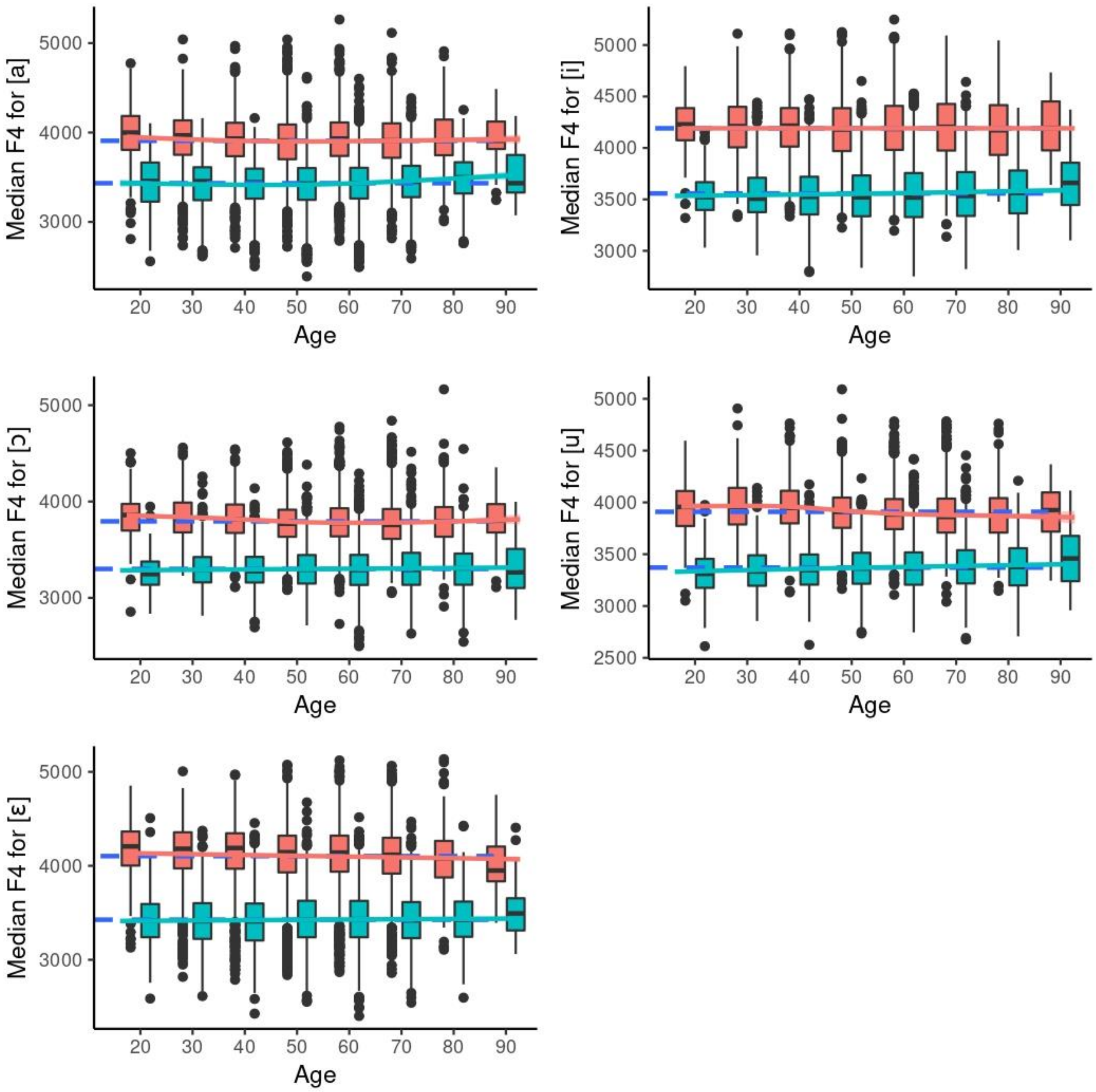

Fig. S6. Vowel formant 4 (F_4) across the lifespan. F_4 (in Hz) is plotted according to age (in years) and sex in the vowels [a], [i], [ɔ], [u] and [ɛ]. Blue boxes indicate values for males, red boxes values for females. For each age group and sex, the bottom and tops of the boxes indicate the bottom and top quartiles, the line inside the box indicates the median, the whiskers indicate the most extreme values inside 1.5 times the interquartile range, and the dots indicate values outside that range.

Supplementary Text

Vowel formant extraction

It is known that automatic vowel formant estimation is error prone and can result in formant numbering errors and contamination from f_o (*1*), the latter, known as *f_o bias*, being particularly difficult to detect and correct. Trained phoneticians show similar patterns of errors as automatic methods (such as the ones used in this study), leading to the conclusion that manual measurement of formant frequencies should not be taken as the gold standard (*81*). f_o bias is known to be a particular concern when voice pitch is high and/or the first formant (F_1) is low (*81*), possibly making formant F_1 in [i] and [u] especially vulnerable since F_1 is lowest in those vowels (figure S3 and table S2). We note that the medians for the vowel formants, including F_1 (figure S3 and table S2), line up closely with expectations based on available data on Icelandic (*96*) and similar vowels in American English (*97*). Nevertheless, given the potential errors in formant estimation, when interpreting results for vowel measures, we recommend considering the range of values and their confidence intervals across a class of measures (e.g., h^2-SNP for F_1 in general), rather than over-interpreting the point estimates for each specific vowel phenotype.

Task demands

Another issue to consider is to what degree the SNP-based heritability estimates and the phenotypic correlations are influenced by details of the experimental design. For instance, reading text requires more cognitive resources than producing isolated vowels. Moreover, the sustained [a] task is more demanding than the isolated (short) vowels both in terms of cognition and respiration. While the short vowel task was preceded by a practice trial, the sustained [a] was elicited without practice and involved a visual prompt (percentage of time completed and a corresponding circle filling up), involving more cognitive load. Sustained vowel phonation – for a duration of 4 seconds in the current study – is also particularly demanding in terms of respiration, and correlates with lung vital capacity in clinical contexts (*98*, *99*). Our phenotypic correlations show that voice pitch variability is correlated with forced vital capacity but only in sustained [a] (table S5). Such differences in elicitation tasks should be kept in mind when interpreting the heritability estimates and the phenotypic correlations.

Table S1 to S11 (separate file)

Table S1: Overview of phenotypes
Table S2: Mean values of phenotypes
Table S3: Correlations between voice and vowel acoustics
Table S4: Age effects on vowel formants
Table S5: Correlations with other phenotypes
Table S6: SNP-based heritability estimates
Table S7: Genetic correlations
Table S8: GWAS lead variants
Table S9: *ABCC9* credible set variants
Table S10: Fixed variant in *ABCC9*
Table S11: Text for reading task

REFERENCES AND NOTES

1. M. C. Corballis, *From Hand to Mouth: The Origins of Language* (Princeton Univ. Press, 2002).

2. W. T. Fitch, *The Evolution of Language* (Cambridge Univ. Press, 2010).

3. C. S. Lai, S. E. Fisher, J. A. Hurst, F. Vargha-Khadem, A. P. Monaco, A forkhead-domain gene is mutated in a severe speech and language disorder. *Nature* **413**, 519–523 (2001).

4. J. den Hoed, S. E. Fisher, Genetic pathways involved in human speech disorders. *Curr. Opin. Genet. Dev.* **65**, 103–111 (2020).

5. R. T. Sataloff, Genetics of the voice. *J. Voice* **9**, 16–19 (1995).

6. E. D. Jarvis, Evolution of vocal learning and spoken language. *Science* **366**, 50–54 (2019).

7. S. C. Vernes, What bats have to say about speech and language. *Psychon. Bull. Rev.* **24**, 111–117 (2017).

8. G. Fant, *Acoustic Theory of Speech Production* (Mouton, 1960).

9. T. Chiba, M. Kajiyama, *The Vowel: Its Nature and Structure* (Phonetic Society of Japan, 1958), vol. 652.

10. I. R. Titze, R. J. Baken, K. W. Bozeman, S. Granqvist, N. Henrich, C. T. Herbst, D. M. Howard, E. J. Hunter, D. Kaelin, R. D. Kent, J. Kreiman, M. Kob, A. Löfqvist, S. McCoy, D. Miller, H. Noé, R. Scherer, J. Smith, B. Story, J. Švec, S. Ternström, J. Wolfe, Toward a consensus on symbolic notation of harmonics, resonances, and formants in vocalization. *J. Acoust. Soc. Am.* **137**, 3005–3007 (2015).

11. J. J. Ohala, An ethological perspective on common cross-language utilization of F0 of voice. *Phonetica* **41**, 1–16 (1984).

12. K. Pisanski, E. C. Mora, A. Pisanski, D. Reby, P. Sorokowski, T. Frackowiak, D. R. Feinberg, Volitional exaggeration of body size through fundamental and formant frequency modulation in humans. *Sci. Rep.* **6**, 34389 (2016).

13. K. Pisanski, V. Cartei, C. McGettigan, J. Raine, D. Reby, Voice modulation: A window into the origins of human vocal control? *Trends Cogn. Sci.* **20**, 304–318 (2016).

14. K. Pisanski, G. A. Bryant, The evolution of voice perception, in *The Oxford Handbook of Voice Studies* (Oxford Univ. Press, 2019), pp. 269–300 .

15. K. Pisanski, A. Oleszkiewicz, J. Plachetka, M. Gmiterek, D. Reby, Voice pitch modulation in human mate choice. *Proc. Biol. Sci.* **285**, 20181634 (2018).

16. T. Aung, D. Puts, Voice pitch: A window into the communication of social power. *Curr. Opin. Psychol.* **33**, 154–161 (2020).

17. D. A. Puts, A. K. Hill, D. H. Bailey, R. S. Walker, D. Rendall, J. R. Wheatley, L. L. Welling, K. Dawood, R. Cárdenas, R. P. Burriss, N. Jablonski, M. Shriver, D. Weiss, A. Lameira, C. Apicella, M. Owren, C. Barelli, M. Glenn, G. Ramos-Fernandez, Sexual selection on male vocal fundamental frequency in humans and other anthropoids. *Proc. Biol. Sci.* **283**, 20152830 (2016).

18. J. Abitbol, P. Abitbol, B. Abitbol, Sex hormones and the female voice. *J. Voice* **13**, 424–446 (1999).

19. C. L. Apicella, D. R. Feinberg, F. W. Marlowe, Voice pitch predicts reproductive success in male hunter-gatherers. *Biol. Lett.* **3**, 682–684 (2007).

20. D. R. Feinberg, B. C. Jones, A. C. Little, D. M. Burt, D. I. Perrett, Manipulations of fundamental and formant frequencies influence the attractiveness of human male voices. *Anim. Behav.* **69**, 561–568 (2005).

21. W. J. Mayew, C. A. Parsons, M. Venkatachalam, Voice pitch and the labor market success of male chief executive officers. *Evol. Hum. Behav.* **34**, 243–248 (2013).

22. D. R. Feinberg, B. C. Jones, M. M. Armstrong, Sensory exploitation, sexual dimorphism, and human voice pitch. *Trends Ecol. Evol.* **33**, 901–903 (2018).

23. L.-J. Boë, T. R. Sawallis, J. Fagot, P. Badin, G. Barbier, G. Captier, L. Ménard, J.-L. Heim, J.-L. Schwartz, Which way to the dawn of speech?: Reanalyzing half a century of debates and data in light of speech science. *Sci. Adv.* **5**, eaaw3916 (2019).

24. K. Pisanski, P. J. Fraccaro, C. C. Tigue, J. J. M. O'Connor, S. Röder, P. W. Andrews, B. Fink, L. M. DeBruine, B. C. Jones, D. R. Feinberg, Vocal indicators of body size in men and women: A meta-analysis. *Anim. Behav.* **95**, 89–99 (2014).

25. J. Rusz, R. Cmejla, T. Tykalova, H. Ruzickova, J. Klempir, V. Majerova, J. Picmausova, J. Roth, E. Ruzicka, Imprecise vowel articulation as a potential early marker of Parkinson's disease: Effect of speaking task. *J. Acoust. Soc. Am.* **134**, 2171–2181 (2013).

26. B. D. Przybyla, Y. Horii, M. H. Crawford, Vocal fundamental frequency in a twin sample: Looking for a genetic effect. *J. Voice* **6**, 261–266 (1992).

27. K. M. Van Lierde, B. Vinck, S. De Ley, G. Clement, P. Van Cauwenberge, Genetics of vocal quality characteristics in monozygotic twins: A multiparameter approach. *J. Voice* **19**, 511–518 (2005).

28. F. Debruyne, W. Decoster, A. Van Gijsel, J. Vercammen, Speaking fundamental frequency in monozygotic and dizygotic twins. *J. Voice* **16**, 466–471 (2002).

29. D. Dediu, E. M. Jennings, D. Van't Ent, S. R. Moisik, G. Di Pisa, J. Schulze, E. J. de Geus, A. den Braber, C. V. Dolan, D. I. Boomsma, The heritability of vocal tract structures estimated from structural MRI in a large cohort of Dutch twins. *Hum. Genet.* **141**, 1905–1923 (2022).

30. S. M. Weinberg, J. Roosenboom, J. R. Shaffer, M. D. Shriver, J. Wysocka, P. Claes, Hunting for genes that shape human faces: Initial successes and challenges for the future. *Orthod. Craniofac. Res.* **22**, 207–212 (2019).

31. M. Šidlauskas, L. Šalomskienė, I. Andriuškevičiūtė, M. Šidlauskienė, Ž. Labanauskas, A. Vasiliauskas, L. Kupčinskas, S. Juzėnas, A. Šidlauskas, Heritability of mandibular cephalometric variables in twins with completed craniofacial growth. *Eur. J. Orthod.* **38**, 493–502 (2016).

32. K. Árnason, *The Phonology of Icelandic and Faroese* (Oxford Univ. Press, 2011).

33. E. V. Ivarsdottir, S. Benonisdottir, G. Thorleifsson, P. Sulem, A. Oddsson, U. Styrkarsdottir, S. Kristmundsdottir, G. A. Arnadottir, G. Thorgeirsson, I. Jonsdottir, G. M. Zoega, U. Thorsteinsdottir, D. F. Gudbjartsson, F. Jonasson, H. Holm, K. Stefansson, Sequence variation at ANAPC1 accounts for 24% of the variability in corneal endothelial cell density. *Nat. Commun.* **10**, 1284 (2019).

34. K. L. Lansford, J. M. Liss, Vowel acoustics in dysarthria: Speech disorder diagnosis and classification. *J. Speech Lang. Hear. Res.* **57**, 57–67 (2014).

35. S. Sapir, L. O. Ramig, J. L. Spielman, C. Fox, Formant centralization ratio: A proposal for a new acoustic measure of dysarthric speech. *J. Speech Lang. Hear. Res.* **53**, 114–125 (2010).

36. D. Reby, K. McComb, Anatomical constraints generate honesty: Acoustic cues to age and weight in the roars of red deer stags. *Anim. Behav.* **65**, 519–530 (2003).

37. G. A. Jonsdottir, G. Einarsson, G. Thorleifsson, S. H. Magnusson, A. F. Gunnarsson, M. L. Frigge, R. S. Gisladottir, U. Unnsteinsdottir, B. Gunnarsson, G. B. Walters, V. Steinthorsdottir, A. Helgadottir, I. Jonsdottir, T. Gislason, H. S. Thorsteinsson, E. Sigurdsson, M. Haraldsson, E. L. Sigurdsson, R. Bjarnason, I. Olafsson, G. Thorgeirsson, P. Sulem, H. Holm, U. Thorsteinsdottir, D. F. Gudbjartsson, G. Bjornsdottir, T. E. Thorgeirsson, H. Stefansson, K. Stefansson, Genetic propensities for verbal and spatial ability have opposite effects on body mass index and risk of schizophrenia. *Intelligence* **88**, 101565 (2021).

38. G. Bjornsdottir, J. G. Halldorsson, S. Steinberg, I. Hansdottir, K. Kristjansson, H. Stefansson, K. Stefansson, The adult reading history questionnaire (ARHQ) in Icelandic: Psychometric properties and factor structure. *J. Learn. Disabil.* **47**, 532–542 (2014).

39. J. Peters, Fluency and speaking fundamental frequency in bilingual speakers of High and Low German, in *Proceedings of the International Congress of Phonetic Sciences* (2019), pp. 1–5.

40. E. H. Magnusdottir, M. Borsky, M. Meier, K. Johannsdottir, J. Gudnason, Monitoring cognitive workload using vocal tract and voice source features. *Period. Polytech. Electr. Eng. Comput. Sci.* **61**, 297–304 (2017).

41. T. F. Yap, J. Epps, E. Ambikairajah, E. H. Choi, Formant frequencies under cognitive load: Effects and classification. *EURASIP J. Adv. Signal Process.* **2011**, 219253 (2011).

42. B. K. Bulik-Sullivan, P.-R. Loh, H. K. Finucane, S. Ripke, J. Yang; Schizophrenia Working Group of the Psychiatric Genomics Consortium, N. Patterson, M. J. Daly, A. L. Price, B. M. Neale, LD Score regression distinguishes confounding from polygenicity in genome-wide association studies. *Nat. Genet.* **47**, 291–295 (2015).

43. D. F. Gudbjartsson, P. Sulem, H. Helgason, A. Gylfason, S. A. Gudjonsson, F. Zink, A. Oddson, G. Magnusson, B. V. Halldorsson, E. Hjartarson, G. Sigurdsson, A. Kong, A. Helgason, G. Masson, O. T. Magnusson, U. Thorsteinsdottir, K. Stefansson, Sequence variants from whole-genome sequencing a large group of Icelanders. *Sci. Data* **2**, 150011 (2015).

44. H. Jónsson, P. Sulem, B. Kehr, S. Kristmundsdottir, F. Zink, E. Hjartarson, M. T. Hardarson, K. E. Hjorleifsson, H. P. Eggertsson, S. A. Gudjonsson, L. Ward, G. A. Arnadottir, E. Helgason, H. Helgason, A. Gylfason, A. Jonasdottir, A. Jonasdottir, T. Rafnar, S. Besenbacher, M. Frigge, S. N. Stacey, O. T. Magnusson, U. Thorsteinsdottir, G. Masson, A. Kong, B. V. Halldorsson, A. Helgason, D. F. Gudbjartsson, K. Stefansson, Whole genome characterization of sequence diversity of 15,220 Icelanders. *Sci. Data.* **4**, 170115 (2017).

45. G. Sveinbjornsson, A. Albrechtsen, F. Zink, S. A. Gudjonsson, A. Oddson, G. Másson, H. Holm, A. Kong, U. Thorsteinsdottir, P. Sulem, D. F. Gudbjartsson, K. Stefansson, Weighting sequence variants based on their annotation increases power of whole-genome association studies. *Nat. Genet.* **48**, 314–317 (2016).

46. J. Bryan, A. Muñoz, X. Zhang, M. Düfer, G. Drews, P. Krippeit-Drews, L. Aguilar-Bryan, ABCC8 and ABCC9: ABC transporters that regulate K^+ channels. *Pflügers Arch.* **453**, 703–718 (2007).

47. T. J. Hund, P. J. Mohler, Differential roles for SUR subunits in K_{ATP} channel membrane targeting and regulation. *Am. J. Physiol. Heart Circ. Physiol.* **300**, H33–H35 (2011).

48. D. A. Puts, S. J. Gaulin, K. Verdolini, Dominance and the evolution of sexual dimorphism in human voice pitch. *Evol. Hum. Behav.* **27**, 283–296 (2006).

49. The Wellcome Trust Case Control Consortium, J. B. Maller, G. McVean, J. Byrnes, D. Vukcevic, K. Palin, Z. Su, J. M. Howson, A. Auton, S. Myers, A. Morris, M. Pirinen, M. Brown, P. Burton, M. Caulfield, A. Compston, M. Farrall, A. Hall, A. Hattersley, A. Hill, C. Mathew, M. Pembrey, J. Satsangi, M. Stratton, J. Worthington, N. Craddock, M. Hurles, W. Ouwehand, M. Parkes, N. Rahman, A. Duncanson, J. Todd, D. Kwiatkowski, N. Samani, S. C. L. Gough, M. I. McCarthy, P. Deloukas, P. Donnelly, Bayesian refinement of association signals for 14 loci in 3 common diseases. *Nat. Genet.* **44**, 1294–1301 (2012).

50. J. P. Pirruccello, M. D. Chaffin, E. L. Chou, S. J. Fleming, H. Lin, M. Nekoui, S. Khurshid, S. F. Friedman, A. G. Bick, A. Arduini, L.-C. Weng, S. H. Choi, A.-D. Akkad, P. Batra, N. R. Tucker, A. W. Hall, C. Roselli, E. J. Benjamin, S. K. Vellarikkal, R. M. Gupta, C. M. Stegmann, D. Juric, J. R. Stone, R. S. Vasan, J. E. Ho, U. Hoffmann, S. A. Lubitz, A. A. Philippakis, M. E. Lindsay, P. T. Ellinor, Deep learning enables genetic analysis of the human thoracic aorta. *Nat. Genet.* **54**, 40–51 (2022).

51. E. Evangelou, H. R. Warren, D. Mosen-Ansorena, B. Mifsud, R. Pazoki, H. Gao, G. Ntritsos, N. Dimou, C. P. Cabrera, I. Karaman, F. L. Ng, M. Evangelou, K. Witkowska, E. Tzanis, J. N. Hellwege, A. Giri, D. R. Velez Edwards, Y. V. Sun, K. Cho, J. M. Gaziano, P. W. F. Wilson, P. S. Tsao, C. P. Kovesdy, T. Esko, R. Mägi, L. Milani, P. Almgren, T. Boutin, S. Debette, J. Ding, F. Giulianini, E. G. Holliday, A. U. Jackson, R. Li-Gao, W.-Y. Lin, J. Luan, M. Mangino, C. Oldmeadow, B. P. Prins, Y. Qian, M. Sargurupremraj, N. Shah, P. Surendran, S. Thériault, N. Verweij, S. M. Willems, J.-H. Zhao, P. Amouyel, J. Connell, R. de Mutsert, A. S. F. Doney, M. Farrall, C. Menni, A. D. Morris, R. Noordam, G. Paré, N. R. Poulter, D. C.

Shields, A. Stanton, S. Thom, G. Abecasis, N. Amin, D. E. Arking, K. L. Ayers, C. M. Barbieri, C. Batini, J. C. Bis, T. Blake, M. Bochud, M. Boehnke, E. Boerwinkle, D. I. Boomsma, E. P. Bottinger, P. S. Braund, M. Brumat, A. Campbell, H. Campbell, A. Chakravarti, J. C. Chambers, G. Chauhan, M. Ciullo, M. Cocca, F. Collins, H. J. Cordell, G. Davies, M. H. de Borst, E. J. de Geus, I. J. Deary, J. Deelen, F. Del Greco M, C. Y. Demirkale, M. Dörr, G. B. Ehret, R. Elosua, S. Enroth, A. M. Erzurumluoglu, T. Ferreira, M. Frånberg, O. H. Franco, I. Gandin, P. Gasparini, V. Giedraitis, C. Gieger, G. Girotto, A. Goel, A. J. Gow, V. Gudnason, X. Guo, U. Gyllensten, A. Hamsten, T. B. Harris, S. E. Harris, C. A. Hartman, A. S. Havulinna, A. A. Hicks, E. Hofer, A. Hofman, J.-J. Hottenga, J. E. Huffman, S.-J. Hwang, E. Ingelsson, A. James, R. Jansen, M.-R. Jarvelin, R. Joehanes, Å. Johansson, A. D. Johnson, P. K. Joshi, P. Jousilahti, J. W. Jukema, A. Jula, M. Kähönen, S. Kathiresan, B. D. Keavney, K.-T. Khaw, P. Knekt, J. Knight, I. Kolcic, J. S. Kooner, S. Koskinen, K. Kristiansson, Z. Kutalik, M. Laan, M. Larson, L. J. Launer, B. Lehne, T. Lehtimäki, D. C. M. Liewald, L. Lin, L. Lind, C. M. Lindgren, Y. Liu, R. J. F. Loos, L. M. Lopez, Y. Lu, L.-P. Lyytikäinen, A. Mahajan, C. Mamasoula, J. Marrugat, J. Marten, Y. Milaneschi, A. Morgan, A. P. Morris, A. C. Morrison, P. J. Munson, M. A. Nalls, P. Nandakumar, C. P. Nelson, T. Niiranen, I. M. Nolte, T. Nutile, A. J. Oldehinkel, B. A. Oostra, P. F. O'Reilly, E. Org, S. Padmanabhan, W. Palmas, A. Palotie, A. Pattie, B. W. J. H. Penninx, M. Perola, A. Peters, O. Polasek, P. P. Pramstaller, Q. T. Nguyen, O. T. Raitakari, M. Ren, R. Rettig, K. Rice, P. M. Ridker, J. S. Ried, H. Riese, S. Ripatti, A. Robino, L. M. Rose, J. I. Rotter, I. Rudan, D. Ruggiero, Y. Saba, C. F. Sala, V. Salomaa, N. J. Samani, A.-P. Sarin, R. Schmidt, H. Schmidt, N. Shrine, D. Siscovick, A. V. Smith, H. Snieder, S. Sõber, R. Sorice, J. M. Starr, D. J. Stott, D. P. Strachan, R. J. Strawbridge, J. Sundström, M. A. Swertz, K. D. Taylor, A. Teumer, M. D. Tobin, M. Tomaszewski, D. Toniolo, M. Traglia, S. Trompet, J. Tuomilehto, C. Tzourio, A. G. Uitterlinden, A. Vaez, P. J. van der Most, C. M. van Duijn, A.-C. Vergnaud, G. C. Verwoert, V. Vitart, U. Völker, P. Vollenweider, D. Vuckovic, H. Watkins, S. H. Wild, G. Willemsen, J. F. Wilson, A. F. Wright, J. Yao, T. Zemunik, W. Zhang, J. R. Attia, A. S. Butterworth, D. I. Chasman, D. Conen, F. Cucca, J. Danesh, C. Hayward, J. M. M. Howson, M. Laakso, E. G. Lakatta, C. Langenberg, O. Melander, D. O. Mook-Kanamori, C. N. A. Palmer, L. Risch, R. A. Scott, R. J. Scott, P. Sever, T. D. Spector, P. van der Harst, N. J. Wareham, E. Zeggini, D. Levy, P. B. Munroe, C. Newton-Cheh, M. J. Brown, A. Metspalu, A. M. Hung, C. J.

O'Donnell, T. L. Edwards, B. M. Psaty, I. Tzoulaki, M. R. Barnes, L. V. Wain, P. Elliott, M. J. Caulfield; Million Veteran Program, Genetic analysis of over 1 million people identifies 535 new loci associated with blood pressure traits. *Nat. Genet.* **50**, 1412–1425 (2018).

52. A. Giri, J. N. Hellwege, J. M. Keaton, J. Park, C. Qiu, H. R. Warren, E. S. Torstenson, C. P. Kovesdy, Y. V. Sun, O. D. Wilson, C. Robinson-Cohen, C. L. Roumie, C. P. Chung, K. A. Birdwell, S. M. Damrauer, S. L. DuVall, D. Klarin, K. Cho, Y. Wang, E. Evangelou, C. P. Cabrera, L. V. Wain, R. Shrestha, B. S. Mautz, E. A. Akwo, M. Sargurupremraj, S. Debette, M. Boehnke, L. J. Scott, J. Luan, J.-H. Zhao, S. M. Willems, S. Thériault, N. Shah, C. Oldmeadow, P. Almgren, R. Li-Gao, N. Verweij, T. S. Boutin, M. Mangino, I. Ntalla, E. Feofanova, P. Surendran, J. P. Cook, S. Karthikeyan, N. Lahrouchi, C. Liu, N. Sepúlveda, T. G. Richardson, A. Kraja, P. Amouyel, M. Farrall, N. R. Poulter; Understanding Society Scientific Group; International Consortium for Blood Pressure; Blood Pressure-International Consortium of Exome Chip Studies, M. Laakso, E. Zeggini, P. Sever, R. A. Scott, C. Langenberg, N. J. Wareham, D. Conen, C. N. A. Palmer, J. Attia, D. I. Chasman, P. M. Ridker, O. Melander, D. O. Mook-Kanamori, P. van der Harst, F. Cucca, D. Schlessinger, C. Hayward, T. D. Spector, M.-R. Jarvelin, B. J. Hennig, N. J. Timpson, W.-Q. Wei, J. C. Smith, Y. Xu, M. E. Matheny, E. E. Siew, C. Lindgren, K.-H. Herzig, G. Dedoussis, J. C. Denny, B. M. Psaty, J. M. M. Howson, P. B. Munroe, C. Newton-Cheh, M. J. Caulfield, P. Elliott, J. M. Gaziano, J. Concato, P. W. F. Wilson, P. S. Tsao, D. R. Velez Edwards, K. Susztak; Million Veteran Program, C. J. O'Donnell, A. M. Hung, T. L. Edwards, Trans-ethnic association study of blood pressure determinants in over 750,000 individuals. *Nat. Genet.* **51**, 51–62 (2019).

53. N. R. Gay, M. Gloudemans, M. L. Antonio, N. S. Abell, B. Balliu, Y. Park, A. R. Martin, S. Musharoff, A. S. Rao, F. Aguet, A. N. Barbeira, R. Bonazzola, F. Hormozdiari, K. G. Ardlie, C. D. Brown, H. K. Im, T. Lappalainen, X. Wen, S. B. Montgomery, Impact of admixture and ancestry on eQTL analysis and GWAS colocalization in GTEx. *Genome Biol.* **21**, 233 (2020).

54. B. V. Halldorsson, H. P. Eggertsson, K. H. S. Moore, H. Hauswedell, O. Eiriksson, M. O. Ulfarsson, G. Palsson, M. T. Hardarson, A. Oddsson, B. O. Jensson, S. Kristmundsdottir, B. D. Sigurpalsdottir, O. A. Stefansson, D. Beyter, G. Holley, V. Tragante, A. Gylfason, P. I. Olason, F. Zink, M. Asgeirsdottir, S. T. Sverrisson, B. Sigurdsson, S. A. Gudjonsson, G. T. Sigurdsson,

G. H. Halldorsson, G. Sveinbjornsson, K. Norland, U. Styrkarsdottir, D. N. Magnusdottir, S. Snorradottir, K. Kristinsson, E. Sobech, G. Thorleifsson, F. Jonsson, P. Melsted, I. Jonsdottir, T. Rafnar, H. Holm, H. Stefansson, J. Saemundsdottir, D. F. Gudbjartsson, O. T. Magnusson, G. Masson, U. Thorsteinsdottir, A. Helgason, H. Jonsson, P. Sulem, K. Stefansson, The sequences of 150,119 genomes in the UK Biobank. *Nature* **607**, 732–740 (2022).

55. P. T. Nelson, G. A. Jicha, W.-X. Wang, E. Ighodaro, S. Artiushin, C. G. Nichols, D. W. Fardo, ABCC9/SUR2 in the brain: Implications for hippocampal sclerosis of aging and a potential therapeutic target. *Ageing Res. Rev.* **24**, 111–125 (2015).

56. N.-Q. Shi, B. Ye, J. C. Makielski, Function and distribution of the SUR isoforms and splice variants. *J. Mol. Cell. Cardiol.* **39**, 51–60 (2005).

57. K. Prüfer, C. de Filippo, S. Grote, F. Mafessoni, P. Korlević, M. Hajdinjak, B. Vernot, L. Skov, P. Hsieh, S. Peyrégne, D. Reher, C. Hopfe, S. Nagel, T. Maricic, Q. Fu, C. Theunert, R. Rogers, P. Skoglund, M. Chintalapati, M. Dannemann, B. J. Nelson, F. M. Key, P. Rudan, Ž. Kućan, I. Gušić, L. V. Golovanova, V. B. Doronichev, N. Patterson, D. Reich, E. E. Eichler, M. Slatkin, M. H. Schierup, A. M. Andrés, J. Kelso, M. Meyer, S. Pääbo, A high-coverage Neandertal genome from Vindija Cave in Croatia. *Science* **358**, 655–658 (2017).

58. K. Prüfer, F. Racimo, N. Patterson, F. Jay, S. Sankararaman, S. Sawyer, A. Heinze, G. Renaud, P. H. Sudmant, C. de Filippo, H. Li, S. Mallick, M. Dannemann, Q. Fu, M. Kircher, M. Kuhlwilm, M. Lachmann, M. Meyer, M. Ongyerth, M. Siebauer, C. Theunert, A. Tandon, P. Moorjani, J. Pickrell, J. C. Mullikin, S. H. Vohr, R. E. Green, I. Hellmann, P. L. F. Johnson, H. Blanche, H. Cann, J. O. Kitzman, J. Shendure, E. E. Eichler, E. S. Lein, T. E. Bakken, L. V. Golovanova, V. B. Doronichev, M. V. Shunkov, A. P. Derevianko, B. Viola, M. Slatkin, D. Reich, J. Kelso, S. Pääbo, The complete genome sequence of a Neanderthal from the Altai Mountains. *Nature* **505**, 43–49 (2014).

59. F. Mafessoni, S. Grote, C. de Filippo, V. Slon, K. A. Kolobova, B. Viola, S. V. Markin, M. Chintalapati, S. Peyrégne, L. Skov, P. Skoglund, A. I. Krivoshapkin, A. P. Derevianko, M. Meyer, J. Kelso, B. Peter, K. Prüfer, S. Pääbo, A high-coverage Neandertal genome from Chagyrskaya Cave. *Proc. Natl. Acad. Sci. U.S.A.* **117**, 15132–15136 (2020).

60. M. Meyer, M. Kircher, M.-T. Gansauge, H. Li, F. Racimo, S. Mallick, J. G. Schraiber, F. Jay, K. Prüfer, C. de Filippo, P. H. Sudmant, C. Alkan, Q. Fu, R. Do, N. Rohland, A. Tandon, M. Siebauer, R. E. Green, K. Bryc, A. W. Briggs, U. Stenzel, J. Dabney, J. Shendure, J. Kitzman, M. F. Hammer, M. V. Shunkov, A. P. Derevianko, N. Patterson, A. M. Andrés, E. E. Eichler, M. Slatkin, D. Reich, J. Kelso, S. Pääbo, A high-coverage genome sequence from an archaic Denisovan individual. *Science* **338**, 222–226 (2012).

61. K. J. Karczewski, L. C. Francioli, G. Tiao, B. B. Cummings, J. Alföldi, Q. Wang, R. L. Collins, K. M. Laricchia, A. Ganna, D. P. Birnbaum, L. D. Gauthier, H. Brand, M. Solomonson, N. A. Watts, D. Rhodes, M. Singer-Berk, E. M. England, E. G. Seaby, J. A. Kosmicki, R. K. Walters, K. Tashman, Y. Farjoun, E. Banks, T. Poterba, A. Wang, C. Seed, N. Whiffin, J. X. Chong, K. E. Samocha, E. Pierce-Hoffman, Z. Zappala, A. H. O'Donnell-Luria, E. V. Minikel, B. Weisburd, M. Lek, J. S. Ware, C. Vittal, I. M. Armean, L. Bergelson, K. Cibulskis, K. M. Connolly, M. Covarrubias, S. Donnelly, S. Ferriera, S. Gabriel, J. Gentry, N. Gupta, T. Jeandet, D. Kaplan, C. Llanwarne, R. Munshi, S. Novod, N. Petrillo, D. Roazen, V. Ruano-Rubio, A. Saltzman, M. Schleicher, J. Soto, K. Tibbetts, C. Tolonen, G. Wade, M. E. Talkowski; Genome Aggregation Database Consortium, B. M. Neale, M. J. Daly, D. G. MacArthur, The mutational constraint spectrum quantified from variation in 141,456 humans. *Nature* **581**, 434–443 (2020).

62. G. Dumas, S. Malesys, T. Bourgeron, Systematic detection of brain protein-coding genes under positive selection during primate evolution and their roles in cognition. *Genome Res.* **31**, 484–496 (2021).

63. J. Blacher, J. A. Staessen, X. Girerd, J. Gasowski, L. Thijs, L. Liu, J. G. Wang, R. H. Fagard, M. E. Safar, Pulse pressure not mean pressure determines cardiovascular risk in older hypertensive patients. *Arch. Intern. Med.* **160**, 1085–1089 (2000).

64. C. G. Nichols, K_{ATP} channels as molecular sensors of cellular metabolism. *Nature* **440**, 470–476 (2006).

65. M. Harakalova, J. J. van Harssel, P. A. Terhal, S. van Lieshout, K. Duran, I. Renkens, D. J. Amor, L. C. Wilson, E. P. Kirk, C. L. Turner, D. Shears, S. Garcia-Minaur, M. M. Lees, A.

Ross, H. Venselaar, G. Vriend, H. Takanari, M. B. Rook, M. A. G. van der Heyden, F. W. Asselbergs, H. M. Breur, M. E. Swinkels, I. J. Scurr, S. F. Smithson, N. V. Knoers, J. J. van der Smagt, I. J. Nijman, W. P. Kloosterman, M. M. van Haelst, G. van Haaften, E. Cuppen, Dominant missense mutations in *ABCC9* cause Cantú syndrome. *Nat. Genet.* **44**, 793–796 (2012).

66. I. Scurr, L. Wilson, M. Lees, S. Robertson, E. Kirk, A. Turner, J. Morton, A. Kidd, V. Shashi, C. Stanley, M. Berry, A. D. Irvine, D. Goudie, C. Turner, C. Brewer, S. Smithson, Cantú syndrome: Report of nine new cases and expansion of the clinical phenotype. *Am. J. Med. Genet. A* **155**, 508–518 (2011).

67. D. K. Grange, H. I. Roessler, C. McClenaghan, K. Duran, K. Shields, M. S. Remedi, N. V. Knoers, J.-M. Lee, E. P. Kirk, I. Scurr, S. F. Smithson, G. K. Singh, M. M. van Haelst, C. G. Nichols, G. van Haaften, Cantú syndrome: Findings from 74 patients in the International Cantú Syndrome Registry. *Am. J. Med. Genet. C Semin. Med. Genet.* **181**, 658–681 (2019).

68. B. W. M. van Bon, C. Gilissen, D. K. Grange, R. C. M. Hennekam, H. Kayserili, H. Engels, H. Reutter, J. R. Ostergaard, E. Morava, K. Tsiakas, B. Isidor, M. Le Merrer, M. Eser, N. Wieskamp, P. de Vries, M. Steehouwer, J. A. Veltman, S. P. Robertson, H. G. Brunner, B. B. A. de Vries, A. Hoischen, Cantú syndrome is caused by mutations in *ABCC9. Am. J. Hum. Genet.* **90**, 1094–1101 (2012).

69. T. M. Olson, A. E. Alekseev, C. Moreau, X. K. Liu, L. V. Zingman, T. Miki, S. Seino, S. J. Asirvatham, A. Jahangir, A. Terzic, K_{ATP} channel mutation confers risk for vein of Marshall adrenergic atrial fibrillation. *Nat. Clin. Pract. Cardiovasc. Med.* **4**, 110–116 (2007).

70. M. Bienengraeber, T. M. Olson, V. A. Selivanov, E. C. Kathmann, F. O'Cochlain, F. Gao, A. B. Karger, J. D. Ballew, D. M. Hodgson, L. V. Zingman, Y.-P. Pang, A. E. Alekseev, A. Terzic, *ABCC9* mutations identified in human dilated cardiomyopathy disrupt catalytic K_{ATP} channel gating. *Nat. Genet.* **36**, 382–387 (2004).

71. M. F. Smeland, C. McClenaghan, H. I. Roessler, S. Savelberg, G. Å. M. Hansen, H. Hjellnes, K. A. Arntzen, K. I. Müller, A. R. Dybesland, T. Harter, M. Sala-Rabanal, C. H. Emfinger, Y.

Huang, S. S. Singareddy, J. Gunn, D. F. Wozniak, A. Kovacs, M. Massink, F. Tessadori, S. M. Kamel, J. Bakkers, M. S. Remedi, M. Van Ghelue, C. G. Nichols, G. van Haaften, *ABCC9*-related Intellectual disability myopathy syndrome is a K_{ATP} channelopathy with loss-of-function mutations in *ABCC9*. *Nat. Commun.* **10**, 4457 (2019).

72. U. Nygren, M. Södersten, H. Falhammar, M. Thoren, K. Hagenfeldt, A. Nordenskjöld, Voice characteristics in women with congenital adrenal hyperplasia due to 21-hydroxylase deficiency. *Clin. Endocrinol. (Oxf.)* **70**, 18–25 (2009).

73. L. Jost, M. Fuchs, M. Loeffler, J. Thiery, J. Kratzsch, T. Berger, C. Engel, Associations of sex hormones and anthropometry with the speaking voice profile in the adult general population. *J. Voice* **32**, 261–272 (2018).

74. J. Steppan, V. Barodka, D. E. Berkowitz, D. Nyhan, Vascular stiffness and increased pulse pressure in the aging cardiovascular system. *Cardiol. Res. Pract.* **2011**, 1–8 (2011).

75. G. F. Mitchell, P. R. Conlin, M. E. Dunlap, Y. Lacourciere, J. M. O. Arnold, R. I. Ogilvie, J. Neutel, J. L. Izzo Jr., M. A. Pfeffer, Aortic diameter, wall stiffness, and wave reflection in systolic hypertension. *Hypertension* **51**, 105–111 (2008).

76. J. Moore, S. Thibeault, Insights into the role of elastin in vocal fold health and disease. *J. Voice* **26**, 269–275 (2012).

77. S. S. Tang, V. Mohad, M. Gowda, S. L. Thibeault, Insights into the role of collagen in vocal fold health and disease. *J. Voice* **31**, 520–527 (2017).

78. A. Hayashi, T. Suzuki, H. Wachi, S. Tajima, T. Nishikawa, S. Murad, S. R. Pinnell, Minoxidil stimulates elastin expression in aortic smooth muscle cells. *Arch. Biochem. Biophys.* **315**, 137–141 (1994).

79. N. R. Wray, S. Ripke, M. Mattheisen, M. Trzaskowski, E. M. Byrne, A. Abdellaoui, M. J. Adams, E. Agerbo, T. M. Air, T. M. Andlauer, S.-A. Bacanu, M. Bækvad-Hansen, A. F. T. Beekman, T. B. Bigdeli, E. B. Binder, D. R. H. Blackwood, J. Bryois, H. N. Buttenschøn, J. Bybjerg-Grauholm, N. Cai, E. Castelao, J. H. Christensen, T.-K. Clarke, J. I. R. Coleman, L.

Colodro-Conde, B. Couvy-Duchesne, N. Craddock, G. E. Crawford, C. A. Crowley, H. S. Dashti, G. Davies, I. J. Deary, F. Degenhardt, E. M. Derks, N. Direk, C. V. Dolan, E. C. Dunn, T. C. Eley, N. Eriksson, V. Escott-Price, F. H. F. Kiadeh, H. K. Finucane, A. J. Forstner, J. Frank, H. A. Gaspar, M. Gill, P. Giusti-Rodríguez, F. S. Goes, S. D. Gordon, J. Grove, L. S. Hall, E. Hannon, C. S. Hansen, T. F. Hansen, S. Herms, I. B. Hickie, P. Hoffmann, G. Homuth, C. Horn, J.-J. Hottenga, D. M. Hougaard, M. Hu, C. L. Hyde, M. Ising, R. Jansen, F. Jin, E. Jorgenson, J. A. Knowles, I. S. Kohane, J. Kraft, W. W. Kretzschmar, J. Krogh, Z. Kutalik, J. M. Lane, Y. Li, Y. Li, P. A. Lind, X. Liu, L. Lu, D. J. MacIntyre, D. F. MacKinnon, R. M. Maier, W. Maier, J. Marchini, H. Mbarek, P. M. Grath, P. M. Guffin, S. E. Medland, D. Mehta, C. M. Middeldorp, E. Mihailov, Y. Milaneschi, L. Milani, J. Mill, F. M. Mondimore, G. W. Montgomery, S. Mostafavi, N. Mullins, M. Nauck, B. Ng, M. G. Nivard, D. R. Nyholt, Paul F. O'Reilly, H. Oskarsson, M. J. Owen, J. N. Painter, C. B. Pedersen, M. G. Pedersen, R. E. Peterson, E. Pettersson, W. J. Peyrot, G. Pistis, D. Posthuma, S. M. Purcell, J. A. Quiroz, P. Qvist, J. P. Rice, B. P. Riley, M. Rivera, S. S. Mirza, R. Saxena, R. Schoevers, E. C. Schulte, L. Shen, J. Shi, S. I. Shyn, E. Sigurdsson, G. B. C. Sinnamon, J. H. Smit, D. J. Smith, H. Stefansson, S. Steinberg, C. A. Stockmeier, F. Streit, J. Strohmaier, K. E. Tansey, H. Teismann, A. Teumer, W. Thompson, P. A. Thomson, T. E. Thorgeirsson, C. Tian, M. Traylor, J. Treutlein, V. Trubetskoy, A. G. Uitterlinden, D. Umbricht, S. Van der Auwera, A. M. van Hemert, A. Viktorin, P. M. Visscher, Y. Wang, B. T. Webb, S. M. Weinsheimer, J. Wellmann, G. Willemsen, S. H. Witt, Y. Wu, H. S. Xi, J. Yang, F. Zhang; eQTLGen; 23andMe, V. Arolt, B. T. Baune, K. Berger, D. I. Boomsma, S. Cichon, U. Dannlowski, E. C. J. de Geus, J. R. De Paulo, E. Domenici, K. Domschke, T. Esko, H. J. Grabe, S. P. Hamilton, C. Hayward, A. C. Heath, D. A. Hinds, K. S. Kendler, S. Kloiber, G. Lewis, Q. S. Li, S. Lucae, P. F. A. Madden, P. K. Magnusson, N. G. Martin, A. M. Mc Intosh, A. Metspalu, O. Mors, P. B. Mortensen, B. Müller-Myhsok, M. Nordentoft, M. M. Nöthen, M. C. O'Donovan, S. A. Paciga, N. L. Pedersen, B. W. J. H. Penninx, R. H. Perlis, D. J. Porteous, J. B. Potash, M. Preisig, M. Rietschel, C. Schaefer, T. G. Schulze, J. W. Smoller, K. Stefansson, H. Tiemeier, R. Uher, H. Völzke, M. M. Weissman, T. Werge, A. R. Winslow, C. M. Lewis, D. F. Levinson, G. Breen, A. D. Børglum, P. F. Sullivan; Major Depressive Disorder Working Group of the Psychiatric Genomics Consortium, Genome-wide association analyses identify 44 risk variants and refine the genetic architecture of major depression. *Nat. Genet.* **50**, 668–681 (2018).

80. M.-T. Lo, D. A. Hinds, J. Y. Tung, C. Franz, C.-C. Fan, Y. Wang, O. B. Smeland, A. Schork, D. Holland, K. Kauppi, N. Sanyal, V. Escott-Price, D. J. Smith, M. O'Donovan, H. Stefansson, G. Bjornsdottir, T. E. Thorgeirsson, K. Stefansson, L. K. McEvoy, A. M. Dale, O. A. Andreassen, C.-H. Chen, Genome-wide analyses for personality traits identify six genomic loci and show correlations with psychiatric disorders. *Nat. Genet.* **49**, 152–156 (2017).

81. C. H. Shadle, H. Nam, D. H. Whalen, Comparing measurement errors for formants in synthetic and natural vowels. *J. Acoust. Soc. Am.* **139**, 713–727 (2016).

82. G. Davies, M. Lam, S. E. Harris, J. W. Trampush, M. Luciano, W. D. Hill, S. P. Hagenaars, S. J. Ritchie, R. E. Marioni, C. Fawns-Ritchie, D. C. M. Liewald, J. A. Okely, A. V. Ahola-Olli, C. L. K. Barnes, L. Bertram, J. C. Bis, K. E. Burdick, A. Christoforou, P. DeRosse, S. Djurovic, T. Espeseth, S. Giakoumaki, S. Giddaluru, D. E. Gustavson, C. Hayward, E. Hofer, M. A. Ikram, R. Karlsson, E. Knowles, J. Lahti, M. Leber, S. Li, K. A. Mather, I. Melle, D. Morris, C. Oldmeadow, T. Palviainen, A. Payton, R. Pazoki, K. Petrovic, C. A. Reynolds, M. Sargurupremraj, M. Scholz, J. A. Smith, A. V. Smith, N. Terzikhan, A. Thalamuthu, S. Trompet, S. J. van der Lee, E. B. Ware, B. G. Windham, M. J. Wright, J. Yang, J. Yu, D. Ames, N. Amin, P. Amouyel, O. A. Andreassen, N. J. Armstrong, A. A. Assareh, J. R. Attia, D. Attix, D. Avramopoulos, D. A. Bennett, A. C. Böhmer, P. A. Boyle, H. Brodaty, H. Campbell, T. D. Cannon, E. T. Cirulli, E. Congdon, E. D. Conley, J. Corley, S. R. Cox, A. M. Dale, A. Dehghan, D. Dick, D. Dickinson, J. G. Eriksson, E. Evangelou, J. D. Faul, I. Ford, N. A. Freimer, H. Gao, I. Giegling, N. A. Gillespie, S. D. Gordon, R. F. Gottesman, M. E. Griswold, V. Gudnason, T. B. Harris, A. M. Hartmann, A. Hatzimanolis, G. Heiss, E. G. Holliday, P. K. Joshi, M. Kähönen, S. L. R. Kardia, I. Karlsson, L. Kleineidam, D. S. Knopman, N. A. Kochan, B. Konte, J. B. Kwok, S. Le Hellard, T. Lee, T. Lehtimäki, S.-C. Li, C. M. Lill, T. Liu, M. Koini, E. London, W. T. Longstreth, O. L. Lopez, A. Loukola, T. Luck, A. J. Lundervold, A. Lundquist, L.-P. Lyytikäinen, N. G. Martin, G. W. Montgomery, A. D. Murray, A. C. Need, R. Noordam, L. Nyberg, W. Ollier, G. Papenberg, A. Pattie, O. Polasek, R. A. Poldrack, B. M. Psaty, S. Reppermund, S. G. Riedel-Heller, R. J. Rose, J. I. Rotter, P. Roussos, S. P. Rovio, Y. Saba, F. W. Sabb, P. S. Sachdev, C. L. Satizabal, M. Schmid, R. J. Scott, M. A. Scult, J. Simino, P. E. Slagboom, N. Smyrnis, A. Soumaré, N. C. Stefanis, D. J. Stott, R. E. Straub, K. Sundet, A. M. Taylor, K. D. Taylor, I. Tzoulaki, C. Tzourio, A.

Uitterlinden, V. Vitart, A. N. Voineskos, J. Kaprio, M. Wagner, H. Wagner, L. Weinhold, K. H. Wen, E. Widen, Q. Yang, W. Zhao, H. H. H. Adams, D. E. Arking, R. M. Bilder, P. Bitsios, E. Boerwinkle, O. Chiba-Falek, A. Corvin, P. L. De Jager, S. Debette, G. Donohoe, P. Elliott, A. L. Fitzpatrick, M. Gill, D. C. Glahn, S. Hägg, N. K. Hansell, A. R. Hariri, M. K. Ikram, J. W. Jukema, E. Vuoksimaa, M. C. Keller, W. S. Kremen, L. Launer, U. Lindenberger, A. Palotie, N. L. Pedersen, N. Pendleton, D. J. Porteous, K. Räikkönen, O. T. Raitakari, A. Ramirez, I. Reinvang, I. Rudan, Dan Rujescu, R. Schmidt, H. Schmidt, P. W. Schofield, P. R. Schofield, J. M. Starr, V. M. Steen, J. N. Trollor, S. T. Turner, C. M. Van Duijn, A. Villringer, D. R. Weinberger, D. R. Weir, J. F. Wilson, A. Malhotra, A. M. McIntosh, C. R. Gale, S. Seshadri, T. H. Mosley, J. Bressler, T. Lencz, I. J. Deary, Study of 300,486 individuals identifies 148 independent genetic loci influencing general cognitive function. *Nat. Commun.* **9**, 2098 (2018).

83. E. Eising, N. Mirza-Schreiber, E. L. de Zeeuw, C. A. Wang, D. T. Truong, A. G. Allegrini, C. Y. Shapland, G. Zhu, K. G. Wigg, M. L. Gerritse, B. Molz, G. Alagöz, A. Gialluisi, F. Abbondanza, K. Rimfeld, M. van Donkelaar, Z. Liao, P. R. Jansen, T. F. M. Andlauer, T. C. Bates, M. Bernard, K. Blokland, M. Bonte, A. D. Børglum, T. Bourgeron, D. Brandeis, F. Ceroni, V. Csépe, P. S. Dale, P. F. de Jong, J. C. DeFries, J.-F. Démonet, D. Demontis, Y. Feng, S. D. Gordon, S. L. Guger, M. E. Hayiou-Thomas, J. A. Hernández-Cabrera, J.-J. Hottenga, C. Hulme, J. Kere, E. N. Kerr, T. Koomar, K. Landerl, G. T. Leonard, M. W. Lovett, H. Lyytinen, N. G. Martin, A. Martinelli, U. Maurer, J. J. Michaelson, K. Moll, A. P. Monaco, A. T. Morgan, M. M. Nöthen, Z. Pausova, C. E. Pennell, B. F. Pennington, K. M. Price, V. M. Rajagopal, F. Ramus, L. Richer, N. H. Simpson, S. D. Smith, M. J. Snowling, J. Stein, L. J. Strug, J. B. Talcott, H. Tiemeier, M. P. van der Schroeff, E. Verhoef, K. E. Watkins, M. Wilkinson, M. J. Wright, C. L. Barr, D. I. Boomsma, M. Carreiras, M.-C. J. Franken, J. R. Gruen, M. Luciano, B. Müller-Myhsok, D. F. Newbury, R. K. Olson, S. Paracchini, T. Paus, R. Plomin, S. Reilly, G. Schulte-Körne, J. B. Tomblin, E. van Bergen, A. J. O. Whitehouse, E. G. Willcutt, B. St Pourcain, C. Francks, S. E. Fisher, Genome-wide analyses of individual differences in quantitatively assessed reading- and language-related skills in up to 34,000 people. *Proc. Natl. Acad. Sci. U.S.A.* **119**, e2202764119 (2022).

84. T. J. Hixon, G. Weismer, J. D. Hoit, *Preclinical Speech Science: Anatomy, Physiology, Acoustics, and Perception* (Plural Publishing, 2018).

85. K. N. Stevens, *Acoustic Phonetics* (MIT Press, 2000), vol. 30.

86. I. R. Helgadóttir, R. Kjaran, A. B. Nikulásdóttir, J. Gudnason, Building an ASR Corpus Using Althingi's Parliamentary Speeches, in *INTERSPEECH* (International Speech Communication Association, 2017), pp. 2163–2167.

87. P. Boersma, Praat, a system for doing phonetics by computer. *Glot Int.* **5**, 341–345 (2001).

88. T. Hastie, R. Tibshirani, Generalized additive models. *Stat. Sci.*, 297–310 (1986).

89. H. P. Eggertsson, H. Jonsson, S. Kristmundsdottir, E. Hjartarson, B. Kehr, G. Masson, F. Zink, K. E. Hjorleifsson, A. Jonasdottir, A. Jonasdottir, I. Jonsdottir, D. F. Gudbjartsson, P. Melsted, K. Stefansson, B. V. Halldorsson, Graphtyper enables population-scale genotyping using pangenome graphs. *Nat. Genet.* **49**, 1654–1660 (2017).

90. A. Kong, G. Masson, M. L. Frigge, A. Gylfason, P. Zusmanovich, G. Thorleifsson, P. I. Olason, A. Ingason, S. Steinberg, T. Rafnar, P. Sulem, M. Mouy, F. Jonsson, U. Thorsteinsdottir, D. F. Gudbjartsson, H. Stefansson, K. Stefansson, Detection of sharing by descent, long-range phasing and haplotype imputation. *Nat. Genet.* **40**, 1068–1075 (2008).

91. A. Buniello, J. A. L. MacArthur, M. Cerezo, L. W. Harris, J. Hayhurst, C. Malangone, A. McMahon, J. Morales, E. Mountjoy, E. Sollis, D. Suveges, O. Vrousgou, P. L. Whetzel, R. Amode, J. A. Guillen, H. S. Riat, S. J. Trevanion, P. Hall, H. Junkins, P. Flicek, T. Burdett, L. A. Hindorff, F. Cunningham, H. Parkinson, The NHGRI-EBI GWAS Catalog of published genome-wide association studies, targeted arrays and summary statistics 2019. *Nucleic Acids Res.* **47**, D1005–D1012 (2019).

92. W. Bai, H. Suzuki, C. Qin, G. Tarroni, O. Oktay, P. M. Matthews, D. Rueckert, Recurrent neural networks for aortic image sequence segmentation with sparse annotations, in *International Conference on Medical Image Computing and Computer-Assisted Intervention* (Springer, 2018), pp. 586–594.

93. J. Y. Dutheil, S. Gaillard, E. H. Stukenbrock, MafFilter: A highly flexible and extensible multiple genome alignment files processor. *BMC Genomics* **15**, 53 (2014).

94. H. Gudbjartsson, G. F. Georgsson, S. A. Gudjonsson, R. Þ. Valdimarsson, J. H. Sigurdsson, S. K. Stefansson, G. Masson, G. Magnusson, V. Palmason, K. Stefansson, GORpipe: A query tool for working with sequence data based on a Genomic Ordered Relational (GOR) architecture. *Bioinformatics* **32**, 3081–3088 (2016).

95. W. McLaren, L. Gil, S. E. Hunt, H. S. Riat, G. R. Ritchie, A. Thormann, P. Flicek, F. Cunningham, The ensembl variant effect predictor. *Genome Biol.* **17**, 122 (2016).

96. C. H. Shadle, H. Nam, D. H. Whalen, Comparing measurement errors for formants in synthetic and natural vowels. *J. Acoust. Soc. Am.* **139**, 713–727 (2016).

97. S. Gunnarsdottir, "Sérhljóð og breytileiki þeirra: Rannsókn á formendatíðni langra og stuttra einhljóða í íslensku" [Vowels and their variability: A study on vowel formants of long and short monophthongs in Icelandic], thesis, University of Iceland, 2012.

98. G. E. Peterson, H. L. Barney, Control methods used in a study of the vowels. *J. Acoust. Soc. Am.* **24**, 175–184 (1952).

99. M. De Letter, P. Santens, M. De Bodt, G. Van Maele, J. Van Borsel, P. Boon, The effect of levodopa on respiration and word intelligibility in people with advanced Parkinson's disease. *Clin. Neurol. Neurosurg.* **109**, 495–500 (2007).

100. G. M. Stegmann, S. Hahn, C. J. Duncan, S. B. Rutkove, J. Liss, J. M. Shefner, V. Berisha, Estimation of forced vital capacity using speech acoustics in patients with ALS. *Amyotroph. Lateral Scler. Front. Degener.* **22**, 14–21 (2021).
